# Molecular Evolution of Chloroplast Genomes of Orchid Species: Insights into Phylogenetic Relationship and Adaptive Evolution

**DOI:** 10.3390/ijms19030716

**Published:** 2018-03-02

**Authors:** Wan-Lin Dong, Ruo-Nan Wang, Na-Yao Zhang, Wei-Bing Fan, Min-Feng Fang, Zhong-Hu Li

**Affiliations:** Key Laboratory of Resource Biology and Biotechnology in Western China, Ministry of Education, College of Life Sciences, Northwest University, Xi’an 710069, China; dongwl@stumail.nwu.edu.cn (W.-L.D.); wangruonan@stumail.nwu.edu.cn (R.-N.W.); 201620812@stumail.nwu.edu.cn (N.-Y.Z.); 201631689@stumail.nwu.edu.cn (W.-B.F.); fangmf@nwu.edu.cn (M.-F.F.)

**Keywords:** adaptive variation, chloroplast genome, molecular evolution, Orchidaceae, phylogenetic relationship

## Abstract

Orchidaceae is the 3rd largest family of angiosperms, an evolved young branch of monocotyledons. This family contains a number of economically-important horticulture and flowering plants. However, the limited availability of genomic information largely hindered the study of molecular evolution and phylogeny of Orchidaceae. In this study, we determined the evolutionary characteristics of whole chloroplast (cp) genomes and the phylogenetic relationships of the family Orchidaceae. We firstly characterized the cp genomes of four orchid species: *Cremastra appendiculata*, *Calanthe davidii*, *Epipactis mairei*, and *Platanthera japonica*. The size of the chloroplast genome ranged from 153,629 bp (*C. davidi*) to 160,427 bp (*E. mairei*). The gene order, GC content, and gene compositions are similar to those of other previously-reported angiosperms. We identified that the genes of *ndhC*, *ndhI*, and *ndhK* were lost in *C. appendiculata*, in that the *ndh I* gene was lost in *P. japonica* and *E. mairei.* In addition, the four types of repeats (forward, palindromic, reverse, and complement repeats) were examined in orchid species. *E. mairei* had the highest number of repeats (81), while *C. davidii* had the lowest number (57). The total number of Simple Sequence Repeats is at least 50 in *C. davidii*, and, at most, 78 in *P. japonica*. Interestingly, we identified 16 genes with positive selection sites (the *psbH*, *petD*, *petL*, *rpl22*, *rpl32*, *rpoC1*, *rpoC2*, *rps12*, *rps15*, *rps16*, *accD*, *ccsA*, *rbcL*, *ycf1*, *ycf2*, and *ycf4* genes), which might play an important role in the orchid species’ adaptation to diverse environments. Additionally, 11 mutational hotspot regions were determined, including five non-coding regions (*ndhB* intron, *ccsA*-*ndhD*, *rpl33*-*rps18*, *ndhE*-*ndhG*, and *ndhF*-*rpl32*) and six coding regions (*rps16*, *ndhC*, *rpl32*, *ndhI*, *ndhK*, and *ndhF*). The phylogenetic analysis based on whole cp genomes showed that *C. appendiculata* was closely related to *C. striata* var. *vreelandii*, while *C. davidii* and *C. triplicate* formed a small monophyletic evolutionary clade with a high bootstrap support. In addition, five subfamilies of Orchidaceae, Apostasioideae, Cypripedioideae, Epidendroideae, Orchidoideae, and Vanilloideae, formed a nested evolutionary relationship in the phylogenetic tree. These results provide important insights into the adaptive evolution and phylogeny of Orchidaceae.

## 1. Introduction

Orchidaceae is the biggest family of monocotyledons and the third largest angiosperm family, containing about five recognized subfamilies (Apostasioideae, Cypripedioideae, Epidendroideae, Orchidoideae, and Vanilloideae) [1], with over 700 genera and 25,000 species [2,3,4]. The orchid species are generally distributed in tropical and subtropical regions in the world, while a few species are found in temperate zones. Many orchid species have important ornamental and flowering values, e.g., their flowers are characterized by labella and a column, and they are attractive to humans [5,6]. In recent years, due to the overexploitation and habitat destruction of orchid species, many wild population resources have become rare and endangered [7]. Presently, some scholars have mainly concentrated on the study of Orchidaceae for their morphology and medicinal value, and research on genomes has been relatively scarce [8,9]. Some studies showed that the two subfamilies, Apostasioideae and Cypripedioideae, were clustered into the two respective genetic clades based partial on chloroplast DNA regions and nuclear markers [4,10]. However, the major phylogenetic relationships among the five orchid subfamilies remain unresolved [11].

In recent years, the fast progress of next-generation sequencing technology has provided a good opportunity for the study of genomic evolution and interspecific relationships of organisms based on large-scale genomic dataset resources, such as complete plastid sequences [12,13]. The chloroplast (cp) is made up of multifunctional organelles, playing a critical role in photosynthesis and carbon fixation [5,14,15,16]. The majority of the cp genomes of angiosperms are circular DNA molecules, ranging from 120 to 160 kb in length, with highly-conserved compositions, in terms of gene content and gene order [17,18,19,20]. Generally, the typical cp genome is composed of a large single copy (LSC) region and a small single copy (SSC) region, which are separated by two copies of inverted repeats (IRa/b) [21,22,23]. Due to its maternal inheritance and conserved structure characteristics [24,25,26,27], the cp genomes can provide abundant genetic information for studying species divergence and the interspecific relationships of plants [28,29,30,31]. For example, based on complete cp genomes, some studies suggested that *Dactylorhiza viridis* diverged earlier than *Dactylorhiza incarnate* [12]; *Lepanthes* is was distinct from *Pleurothallis* and *Salpistele* [13]. In addition, some researchs based on one nuclear region (*ITS*-*1*) and five chloroplast DNA fragment variations revealed that *Bolusiella talbotii* and the congeneric *B. iridifolia* were clustered into an earlier diverged lineage [10]. However, up to now, the phylogenetic relationships of some major taxons (e.g., *Cremastra* and *Epipactis*) in the Orchidaceae family remain unclear. 

In this study, the complete cp genomes of four orchid species (*Cremastra appendiculata*, *Calanthe davidii*, *Epipactis mairei*, and *Platanthera japonica*) were first assembled and annotated. Following this, we analyzed the differences in genome size, content, and structure, and the inverted repeats (IR) contraction and expansion, identifying the sequence divergence, along with variant hotspot regions and adaptive evolution through combination with other available orchid cp genomes. In addition, we also constructed the evolutionary relationships of the Orchidaceae family, based on the large number of cp genome datasets.

## 2. Results

### 2.1. The Chloroplast Genome Structures

**Figure 1:**
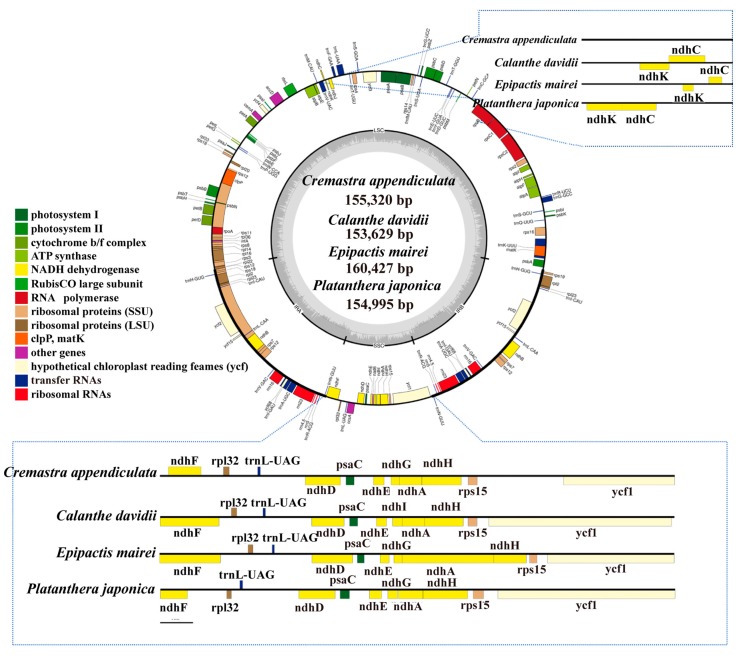
Chloroplast genome maps of the four orchid species. Gene locations outside of the outer rim are transcribed in the counter clockwise direction, whereas genes inside are transcribed in the clockwise direction. The colored bars indicate known different functional groups. The dashed gray area in the inner circle shows the proportional GC content of the corresponding genes. LSC, SSC and IR are large single-copy region, small single-copy region, and inverted repeat region, respectively.

In this study, the cp genomes of four species displayed a typical quadripartite structure and similar lengths, containing a pair of inverted repeats IR regions (IRa and IRb), one large single-copy (LSC) region, and one small single-copy (SSC) region (Figure 1, Table 2). The cp genome size ranged from 153,629 bp in *C. davidii* to 160,427 bp in *E. mairei*, with *P. japonica* at 154,995 bp and *C. appendiculata* at 155,320 bp. The length of LSC ranged from 85,979 bp (*P. japonica*) to 88,328 bp (*E. mairei*), while the SSC length and IR length ranged from 13,664 bp (*P. japonica*) to 18,513 bp (*E. mairei*), and from 25,956 bp (*C. davidii*) to 27,676 bp (*P. japonica*). In the four species, the GC contents of the LSC and SSC regions (about 34% and 40%) were lower than those of the IR regions (about 43%) (Table 1). There were 37 tRNA genes and eight rRNA genes that were identified in each orchid cp genome, but there were some differences in terms of protein-coding genes. In *C. davidii*, we annotated 86 protein-coding genes. There were no *ndhC*, *ndhI*, and *ndhK* genes in *C. appendiculata*. In *P. japonica* and *E. mairei*, the *ndhI* gene was lost (Table 1 and Table 2). Fourteen out of the seventeen genes contained a single intron, while three (*clpP*, *ycf3*, and *rps12*) had two introns (Table 2).

### 2.2. Repeat Structure and Simple Sequence Repeats 

**Figure 2:**
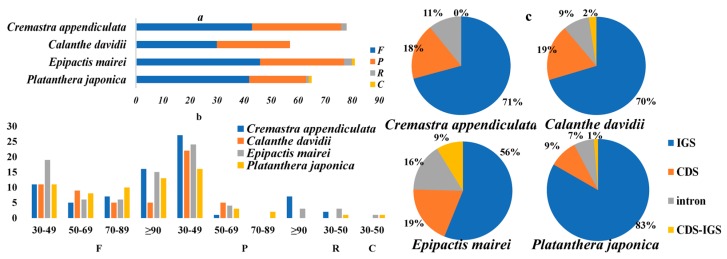
Maps of repeat sequence analyses. Repeat sequences in *C. appendiculata*, *C. davidii*, *E. mairei*, and *P. japonica* chloroplast genomes. (**a**) Number of the four repeat types, F, P, R, and C, indicate the repeat type (F: forward, P: palindrome, R: reverse, and C: complement, respectively). (**b**) Frequency of the four repeat types by length. (**c**) Repeat distribution among four different regions: IGS: intergenic spacer, CDS: coding sequence, intron and CDS-IGS part in CDS and part in IGS.

**Figure 3:**
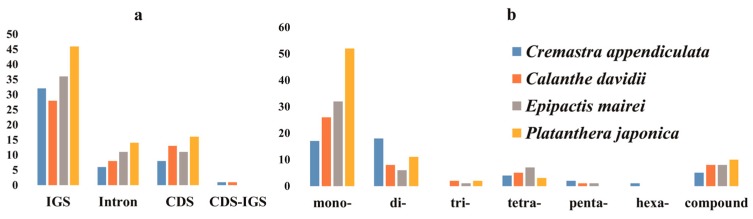
The distribution maps of simple sequence repeats (SSR) in *C. appendiculata*, *C. davidii*, *E. mairei*, and *P. japonica* chloroplast genomes. (**a**) Classification of SSRs in four orchid species. IGS, intergenic spacer; CDS, coding sequence, CDS-IGS, part in CDS and part in IGS. (**b**) Classification of SSRs by repeat type. mono-, mononucleotides; di-, dinucleotides; tri-, trinucleotides; tetra-, tetranucleotides; penta-, pentanucleotides; and hexa-, hexanucleotides.

Repeats in cp genomes were analyzed using REPuter (Figure 2a and Table S2). *E. mairei* had the greatest number, including 46 forward, 31 palindromic, three reverse repeats, and 1 complement repeat. This was followed by *C. appendiculata* with 43 forward, 33 palindromic, and 2 reverse repeats. *P. japonica* had 42, 21, 1, and 1 forward, palindromic, reverse, and complement repeats. *C. davidii* had the least number, with only 30 forward and 27 palindromic repeats. The comparison analyses revealed that most of the repeats were 30–90 bp, and that the longest repeats, with a length of 309 bp, were detected in the *E. mairei* cp genome (Figure 2b). Most of the repeats were distributed in non-coding regions. There were 9% repeats in coding sequence and intergenic spacer parts (CDS-IGS) in *E. mairei*, but none in *C. appendiculata* (Figure 2c). The highest number of tandem repeats was 53 in *E. mairei*, and the lowest was 29 in *C. davidii* (Table S3). The total number of SSRs was 51 in *C. appendiculata*, 50 in *C. davidii*, 58 in *E. mairei*, and 78 in *P. japonica* (Table S4). Only one six compound, SSR, was found in *C. appendiculata* (Figure 3a). A large proportion of SSRs were found in the LSC region, and we did not identify C/G mononucleotide repeats, while the majority of the dinucleotide repeat sequences were comprised of AT/TA repeats (Figure 3b). 

### 2.3. IR Contraction and Expansion

**Figure 4:**
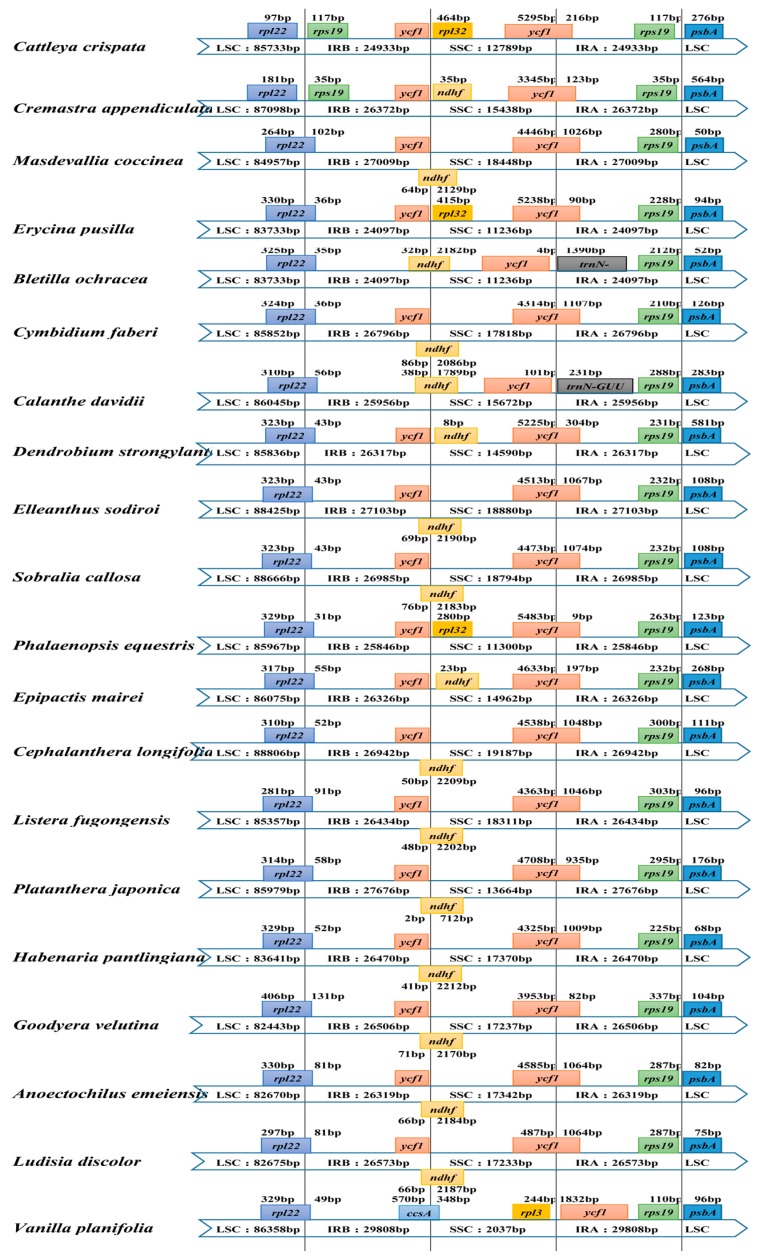
Comparison of the borders of LSC, SSC, and IR regions in 20 orchid complete chloroplast genomes.

We examined the differences between inverted repeat and single-copy (IR/SC) boundary regions among 20 orchid genera, which were classified into several different types (Figure 4). First, the *rps19* gene crossed the large single-copy and inverted repeat b (LSC/IRb) regions within the two parts for eighteen Orchidaceae genera. In *C. crispate* and *C. appendiculata*, the *rps19* gene existed only in the IRb region. Second, in 12 genera, the *ndhF* gene and the *ycf1* pseudogene overlapped in the IRb/SSC region. In *C. appendiculata* and *Dendrobium strongylanthum*, the *ndhF* gene was complete in the SSC region, 8–35 bp away from the IRb region. In *C. crispate*, *E. pusilla*, and *Phalaenopsis equestris*, the *rpl32* gene was in the SSC region instead of the *ndhF* gene, 280–464 bp away from the IRb region. For the 17 genera mentioned above, the *ycf1* gene crossed the SSC/IRa region. In *C. edavidii* and *Bletilla ochracea*, the *ndhF* gene crossed the IRb/SSC region, and the *ycf1* gene was complete in the SSC region, 101 and 4 bp away from the IRa region. The *trnH*-*GUG* genes were all located in the LSC region, which was 231 to 1390 bp away from the LSC/IRa boundary. Most specifically, in *Vanilla planifolia*, the *ccsA* gene crossed the IRb/SSC region, as we did not find the *ndhF* and *ycf1* genes where they should be. The SSC/IRa borders were located between the *rpl32* and *ycf1* genes. Thirdly, all 20 genera had the same IRa/LSC borders: the *rps19* gene in the IRa region and the *psbA* gene in the LSC region.

### 2.4. Sequence Divergence and Mutational Hotspot

**Figure 5:**
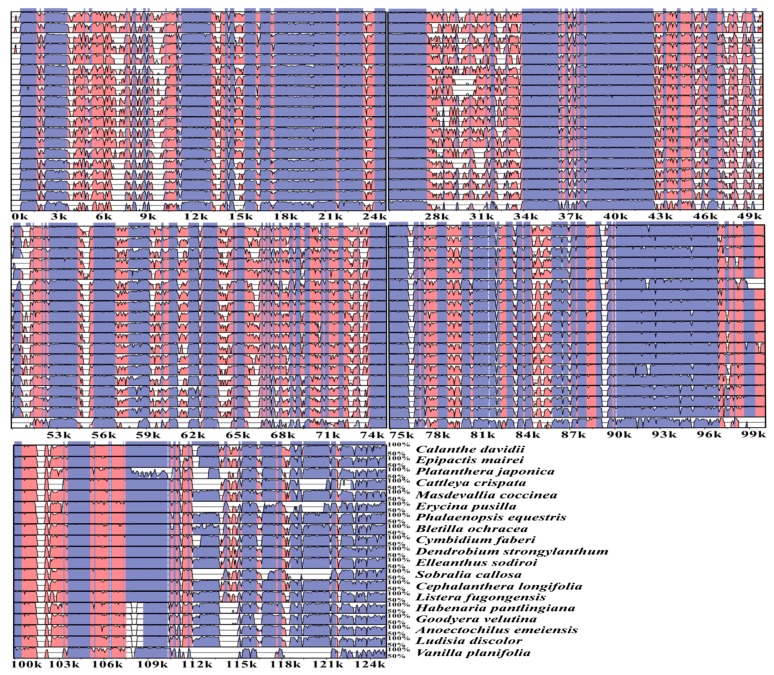
Sequence alignment of chloroplast genomes of 20 orchid species. Sequence identity plot comparing the chloroplast genomes with *C. appendiculata* as a reference using mVISTA. The red color-coded as intergenic spacer regions. The blue color-coded as gene regions. A cut-off of 70% identity was used for the plots, and the *Y*-scale represents the percent identity between 50% and 100%.

**Figure 6:**
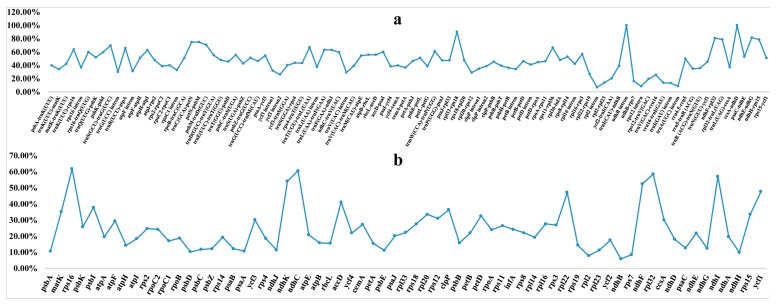
Percentages of variable sites in homologous regions across the 20 orchids with complete chloroplast genomes. (**a**) The introns and spacers (IGS); and (**b**) protein coding sequences (CDS).

The whole chloroplast genome sequences of *C. appendiculata*, *C. davidii*, *E. mairei*, and *P. japonica* were compared to 16 other species, using mVISTA [32] (Figure 5 and Figure 6, and Table S5). The comparison analyses showed a high sequence similarity across the cp genomes, with a sequence identity of 82.0%. Interestingly, the proportions of variability in the non-coding regions (introns and intergenic spacers) ranged from 6.77% to 100% with a mean value of 45.97%, i.e., values that are twice as high as in the coding regions (where the range was from 5.80% to 61.76% with a mean value of 24.68%). Five regions within the non-coding regions (*ndhB* intron, *ccsA*-*ndhD*, *rpl33*-*rps18*, *ndhE*-*ndhG*, and *ndhF*-*rpl32*) and six regions within the coding parts (*rps16*, *ndhC*, *rpl32*, *ndhI*, *ndhK*, and *ndhF*) showed greater levels of variations (percentage of variability >80% and 50%, respectively). In particular, the *ndhB* intron and *ccsA*-*ndhD* showed a variable percentage of 100%.

**Figure 7:**
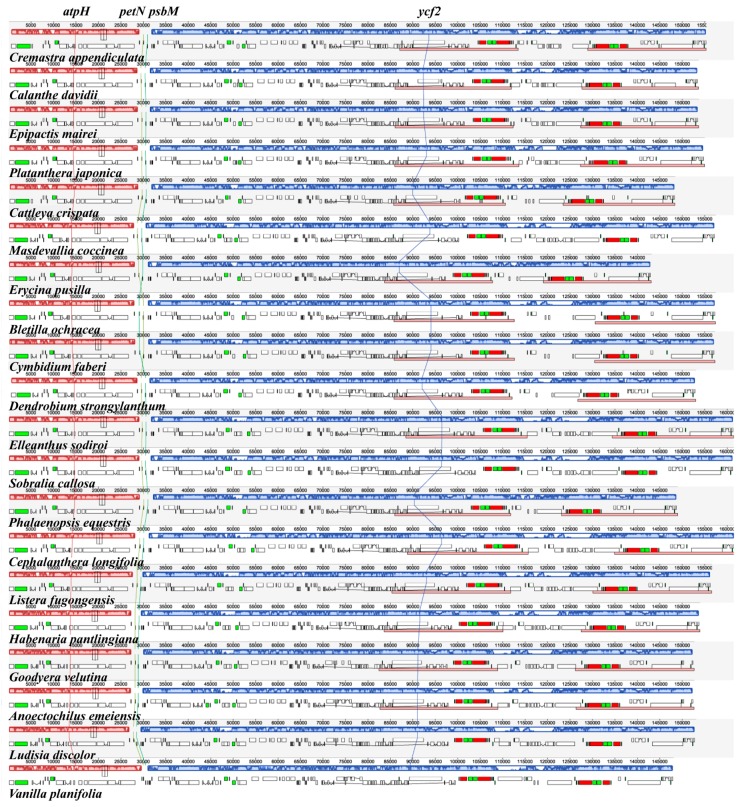
MAUVE genome alignments of the 20 orchid chloroplast genomes, with *C. appendiculata* set as a reference genome. The corresponding colored boxes indicate locally-collinear blocks, which present homologous gene clusters. The red vertical line is the location of *atpH* gene. The yellow vertical line is the location of *petN* gene. The green vertical line is the location of *psbM* gene. The blue vertical line is the location of *ycf2* gene.

In addition, we performed a MAUVE [33] alignment of the 20 orchid chloroplast genomes. The *C. appendiculata* genome is shown at the top as the reference genome (Figure 7). These species maintained a consistent sequence order in most of the genes. However, in *B. ochracea* and *C. faberi*, the *psbM* gene was in front of the *petN*, while the others were upside-down. *Bletilla* and *Cymbidium* actually had the nearest relationship.

### 2.5. Gene Selective Analysis

We compared the rate of nonsynonymous (dN) and synonymous (dS) substitutions for 68 common protein-coding genes between *C. appendiculata*, *C. davidii*, *E. mairei*, and *P. japonica* with 16 other Orchidaceae species (Table S6). Sixteen genes with positive selection sites were identified (Table S7). These genes included one subunit of the photosystem II gene (*psbH*), two genes for cytochrome b/f complex subunit proteins (*petD* and *petL*), two genes for ribosome large subunit proteins (*rpl22* and *rpl32*), two DNA-dependent RNA polymerase genes (*rpoC1* and *rpoC2*), three genes for ribosome small subunit proteins (*rps12*, *rps15*, and *rps16*), and *accD*, *ccsA*, *rbcL*, *ycf1*, *ycf2*, and *ycf4* genes. Interestingly, the *ycf1* gene possesses 13 and 15 positive selective sites, followed by *accD* (8, 10), *rbcL* (4, 7), *ycf2* (2, 3), *rpoC1* (2, 4), *rpoC2* (1, 2), *rpl22* (1, 2), *rps16* (1, 2), *rpl32* (1, 1), *rps12* (1, 1), *ccsA* (0, 2), *petD* (0, 1), *petL* (0, 1), *psbH* (0, 1), and *ycf4* (0, 1). What is more, the likelihood ratio tests (LRTs) of variables under different models were compared in the site-specific models, M0 vs. M3, M1 vs. M2 and M7 vs. M8, in order to support the sites under positive selection (*p* < 0.01) (Table S7).

### 2.6. Phylogenetic Relationship

**Figure 8:**
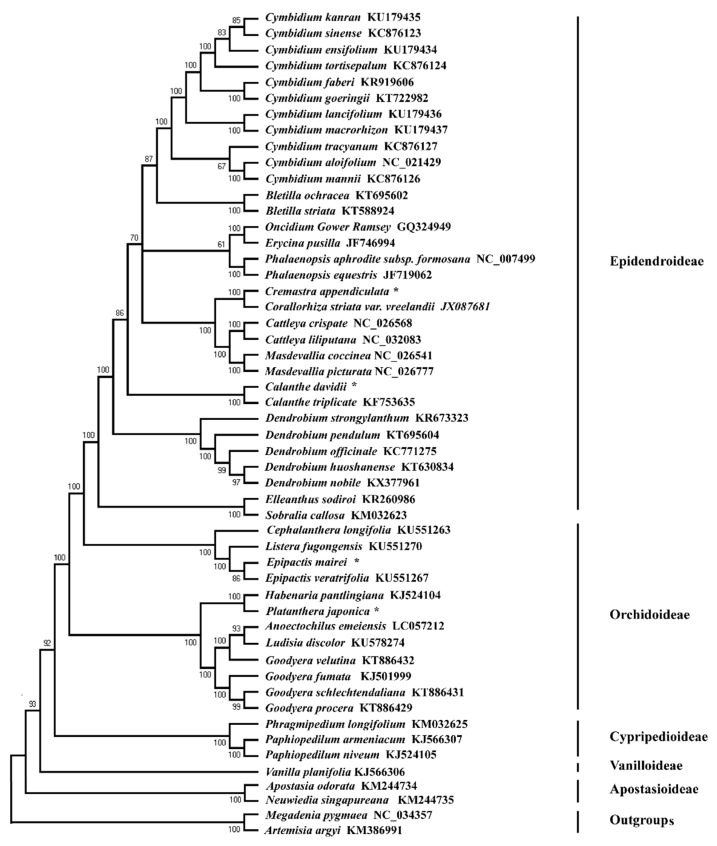
Cladogram of 54 nucleotide sequences of complete chloroplast genomes of orchid species based on the GTRGAMMA model with maximum likelihood (ML) analysis. * The newly generated chloroplast genomes of orchid species.

In this study, the maximum likelihood (ML) analysis suggested that *C. appendiculata* and the congeneric *C. davidii* clustered into the Epidendroideae subfamily clade with high bootstrap support, and that *E. mairei* and *P. japonica* clustered into Orchidoideae subfamily (Figure 8). Interestingly, five subfamilies of Orchidaceae, Apostasioideae, Cypripedioideae, Epidendroideae, Orchidoideae, and Vanilloideae have a nested evolutionary relationship in the ML tree. Meanwhile, *C. appendiculata* was closely-related to *C. striata* var. *vreelandii*, *C. davidii*, and *C. triplicate*, which formed a small evolutionary clade with a high bootstrap. *P. japonica* and *Habenaria pantlingiana* had a relatively-closer affinity in the Orchidoideae subfamily.

## 3. Discussion

### 3.1. Sequence Variation

In this study, we first determined the whole chloroplast genomes of four orchid species. The cp genome size of *C. davidii* was shorter than that of others, which might be the result of the expansion and contraction of the border positions between the IR and SC regions [21,22,23]. In addition, the GC contents of the LSC and SSC regions in all the orchid species were much lower than those of the IR regions, which possibly resulted from four rRNA genes (*rrn16*, *rrn23*, *rrn4.5*, and *rrn5*) sequences in the IR regions. In addition, we identified some obvious differences in the protein-coding genes for the orchid chloroplast genomes, despite that the cp genomes of land plants are generally considered to be highly conserved [34]. Interestingly, there were no *ndhC*, *ndhI*, and *ndhK* genes in *C. appendiculata*. In *P. japonica* and *E. mairei*, the *ndhI* gene was lost. Previous studies also found that some orchid species had lost the *ndh* gene, which encodes the subunits of the nicotinamide-adenine dinucleotid (NADH) dehydrogenase-like complex proteins [35,36,37]. The loss of this gene might have hindered cyclic electron flow around photosystem I and affected the plant photosynthesizing [35,36,37,38]. In addition, some studies suggested that the different Orchidaceae species harbored a variable loss or retention of *ndh* genes [35]. For example, *Cymbidium* has the *ndhE*, *ndhJ*, and *ndhC* genes [39], and *Oncidium* has the *ndhB* gene [40]. Nevertheless, the mechanisms that underlie the variable loss or retention of *ndh* genes in orchids remain unclear [11,41].

In addition, we identified 233 SSRs in four orchid species (*C. appendiculata*, *C. davidii*, *E. mairei*, and *P. japonica*); 77.68% of SSRs were distributed in the IGS and intron regions. Generally, microsatellites consist of 1–6 nucleotide repeat units, which are widely distributed across the entire genome and have a great influence on genome recombination and rearrangement [42,43]. The large amount of SSRs also have been identified in *Forsythia suspense* [44], *Dendrobium nobile*, *Dendrobium officinale*, and so on [45]. The majority of these SSRs consisted of mono- and di-nucleotide repeats. Tri-, tetra-, and penta-nucleotide repeat sequences were detected at a much lower frequency in these orchid species and in other organisms [46,47]. 

Meanwhile, our analyses revealed that the mutational hotspots among orchid genera were highly variable. A diversity of IR contraction and expansion, along with the high level of mutational hotspots, revealed that Orchidaceae had experienced a complex evolution process. Interestingly, in the orchid species, the two IR regions were less divergent than the LSC and SSC regions. Five regions within the non-coding regions (*ndhB* intron, *ccsA*-*ndhD*, *rpl33*-*rps18*, *ndhE*-*ndhG*, and *ndhF*-*rpl32*) and six regions within the coding regions (*rps16*, *ndhC*, *rpl32*, *ndhI*, *ndhK*, and *ndhF*) showed greater levels of variations (percentage of variability >80% and 50%, respectively). These regions can be used as potential DNA barcodes for the further study of phylogenetic relationships, species identification, and population genetics.

### 3.2. Adaptive Evolution

We used the site-specific model (seqtype = 1, model = 0, NSsites = 0, 1, 2, 3, 7, 8), one of the codon substitution models, to estimate the selection pressure [48]. Sixteen genes with positive selection sites were identified in these orchid species. These genes included one subunit of the photosystem II gene (*psbH*), two genes for cytochrome b/f complex subunit proteins (*petD* and *petL*), two genes for ribosome large subunit proteins (*rpl22* and *rpl32*), two DNA-dependent RNA polymerase genes (*rpoC1* and *rpoC2*), three genes for ribosome small subunit proteins (*rps12*, *rps15*, and *rps16*), and *accD*, *ccsA*, *rbcL*, *ycf1*, *ycf2*, and *ycf4* genes. We found that the genes with positive selection sites can be divided into four categories: Subunits of photosystem (*psbH* and *ycf4*), subunits of cytochrome (*petD*, *petL*, and *ccsA*), subunits of ribosome (*rpl22*, *rpl32*, *rps12*, *rps15*, and *rps16*) and others (*rpoC1*, *rpoC2*, *accD*, *rbcL*, *ycf1*, and *ycf2*). The plastid *accD* gene, which encodes the β-carboxyl transferase subunit of acetyl-CoA carboxylase, is an essential and required component for plant leaf development [49,50,51,52,53]. In this study, 10 positively-selected sites were identified in *accD* genes for orchid species, suggesting that the *accD* gene played a possible pivotal role in the adaptive evolution of orchids. What is more, the *ycf1* gene is also essential for almost all plant lineages [5,54], except for Gramineae, which lost the *ycf1* gene in its cp genomes [55]. Additionally, *ycf1* is one of the largest cp genes, encoding a component of the chloroplast’s inner envelope membrane protein translocon [56]. This gene, which is also highly variable in terms of phylogenetic information at the level of species, has also been shown to be subject to positive selection with 15 sites, as has also been identified in many plant lineages [57,58,59]. In addition, we found that the *rbcL* gene possessed seven sites under positive selection in orchid species. Generally, *rbcL* is the gene for the Rubisco large subunit protein, and as the result of enzymatic activity of Rubisco, which is an important component as a modulator of photosynthetic electron transport [60,61]. Current research has revealed that positive selection of the *rbcL* gene in land plants may be a common phenomenon [62]. Additionally, the *rbcL* gene is also widely used in the phylogenetic analysis of land plants [63]. In conclusion, these results showed that multiple factors, several of them interconnected (positive selection, heterogeneity environments), have possibly contributed to orchid diversification and adaptation. For example, some positively-selected sites that were identified (e.g., *rbcL*, *ycf1*, and *accD*) were associated in a significant manner with environment adaptation, including factors such as temperature, light, humidity, and atmosphere [49]. Additionally, epiphytism in orchid species is a key innovation which should help generate and maintain high levels of plant diversity. On the other hand, the tropical distributions of orchid species might have increased the rates of speciation relative to those outside of the tropics as a result of more stable climates (e.g., the lack of glaciation and suitable temperatures), the greater habitat area, and together, this possibly provided a greater opportunity for the co-evolution of plants and their mutualists, and for greater adaptation [49,58,59].

### 3.3. Phylogenetic Relationship

In this study, the maximum likelihood (ML) tree obtained high bootstrap support values, which had 33 nodes with 100% bootstrap support, with 36 of the 46 nodes having values ≥95%. The phylogenetic analyses based on complete cp genomes, suggested that five subfamilies of Orchidaceae (Apostasioideae, Cypripedioideae, Epidendroideae, Orchidoideae, and Vanilloideae) have a nested evolutionary relationship (Figure 8). Apostasioideae is the earliest diverging subfamily of orchids. Some recent molecular studies have shown that the five subfamilies had formed their respective five monophylies [11,41,49]. The generic relationships of the five subfamilies found in our analyses are basically congruent with those of recent studies. However, our finding that Orchidoideae is a nested subfamily is different from the studies of Kim et al. [41] and Givnish et al. [49]. They reconstructed ML trees using the concatenated coding sequences of plastid genes, resulting in large amounts of missing data for these orchid taxa. In this study, we sampled these newly orchid species (*C. longifolia*, *L. fugongensis*, *E. mairei*, and *E. veratrifolia*) to construct a more widespread Orchidaceae phylogenetic tree, through which we obtained the different species relationships. However, some molecular phylogenetic studies, to date, have failed to identify the placement of Cypripedioideae and Vanilloideae [8,25,64,65]. Recently, Givnish et al. [49] and Niu et al. [11] reconstructed ML trees from 39 and 53 orchids species, respectively, using the sequence variations in 75 genes and 67 genes from the plastid genomes. Their results showed that five orchid subfamilies clustered into the five monophyletic clades: Epidendroideae–Orchidoideae–Cypripedioideae–Vanilloideae–Apostasioideae. However, the current study found that *C. appendiculata* and the congeneric *C. davidii* clustered into the Epidendroideae subfamily clade, and that *E. mairei* and *P. japonica* clustered into the Orchidoideae subfamily. These results were largely consistent with traditional morphological evidence [66,67,68]. However, the inconsistent phylogenetic relationships for the five subfamilies may be due to the differences in the collected samples used in different studies [11,49,64,65], which need to be further explored by sampling a much higher number of orchid species.

## 4. Materials and Methods

### 4.1. Plant Material, DNA Extraction, Library Construction, and Sequencing

Fresh leaf tissues were collected from *Cremastra appendiculata*, *Calanthe davidii*, *Epipactis mairei*, and *Platanthera japonica* in the Qinling Mountains, Shaanxi Province, China. The leaves were cleaned and preserved in a −80 °C refrigerator at Northwest University. The voucher specimens of the four species materials were deposited into the Northwest University Herbarium (NUH). The total genomic DNA was isolated using the modified Cetyltrimethyl Ammonium Bromide (CTAB) method [69], which added the EDTA buffer (Amresco, Washington, DC, USA) (1.0 mol/L Tris-HCl (Amresco, Washington, DC, USA) (pH 8.0), 0.5 mol/L EDTA-Na_2_ (Amresco, Washington, DC, USA), 5.0 mol/L NaCl) solution before isolating the high-quality DNA with the CTAB solution (1.0 mol/L Tris-HCl (pH 8.0), 0.5 mol/L EDTA-Na_2_, 2% CTAB). Following this, we constructed a pair-end (PE) library with 350 bp insert size fragments using TruSeq DNA sample preparation kits (Sangon, Shanghai, China). Subsequently, we sequenced at least 4.5 GB of clean data for each orchid species. The detailed next-generation sequencing was conducted on the Illumina Hiseq 2500 platform by Sangon Biotech (Shanghai, China).

### 4.2. Chloroplast Genome Assembly and Annotation

First, we used the software, NGSQCToolkit v2.3.3 [70], to trim the low-quality reads. After removing the low-quality sequences, the clean reads were assembled using MIRA v4.0.2 [70] and MITObim v1.8 [71] with the cp genome of a closely-related species, *Dendrobium nobile* (KX377961), as reference. The programs, DOGMA (http://dogma.ccbb.utexas.edu/) [72] and Geneious v8.0.2 [73] were used to annotate the chloroplast genome. Finally, we obtained four high-quality, complete chloroplast genome sequences. The Circle maps of the four species were drawn using OGDRAW v1.1 [74].

### 4.3. Repeat Sequence Analyses

The REPuter program (Available online: https://bibiserv.cebitec.uni-bielefeld.de/reputer/manual.html) was used to identify repeats, including forward, reverse, palindrome, and complement sequences. The maximum computed repeats and the minimal repeat size were limited to 50 and 30, respectively, with a Hamming distance equal to 3 [75]. The tandem repeats finder welcome page (http://tandem.bu.edu/trf/trf.html) was used to identify tandem repeats sequences [76]. The alignment parameters match, mismatch, and indels, were 2, 7, and 7, respectively. The minimum alignment score to report repeat, maximum period size and maximum TR array Size (bp, millions) are limited to 80, 500, and 2, respectively. A Perl script MISA (MIcroSAtellite identification tool, http://pgrc.ipk-gatersleben.de/misa/) was used to search for simple sequence repeat (SSR or microsatellite) loci in the chloroplast genomes [77]. Tandem repeats of 1–6 nucleotides were viewed as microsatellites. The minimum number of repeats were set to 10, 5, 4, 3, 3, and 3, for mono-, di-, tri-, tetra-, penta-, and hexanucleotides, respectively.

### 4.4. Genome Structure and Mutational Hotspot

In order to compare the genome structures and divergence hotspots in a broad manner, we used 16 cp genomes (available in Genbank https://www.ncbi.nlm.nih.gov/) representing each orchid genus, and added the four newly-sequenced ones (Table 3). The boundaries between the IR and SC regions of *C. appendiculata*, *C. davidii*, *E. mairei*, and *P. japonica* and other 16 sequences were compared and analyzed. Meanwhile, the whole-genome alignment of the chloroplast genomes of the 20 species were performed and plotted using the mVISTA program [32]. Following this, we selected the regions within non-coding and coding regions that had a greater level of variation (percentage of variability >80% and 50%, respectively) as mutational hotspots. The formula was as follows: percentage of variable = (number of nucleotide substitutions + the number of indels)/(the length of aligned sites − the length of indels + the number of indels) × 100%.

### 4.5. Gene Selective Pressure Analysis

The codon substitution models in the Codeml program, PAML3.15 [46] were used for calculating the non-synonymous (dN) and synonymous (dS) substitution rates, along with their ratios (*ω* = dN/dS). We analyzed all CDS gene regions, except *ndh*, due to there being too many losses there. These unique CDS gene sequences were separately extracted and aligned using Geneious v8.0.2 [73]. A maximum likelihood phylogenetic tree was built based on the complete cp genomes of the 20 species using RAxML [78]. We used the site-specific model (seqtype = 1, model = 0, NSsites = 0, 1, 2, 3, 7, 8) to estimate the selection pressure [79]. This model allowed the *ω* ratio to vary among sites, with a fixed *ω* ratio in all the branches. Comparing the site-specific model, M1 (nearly neutral) vs. M2 (positive selection), M7 (β) vs. M8 (β and *ω*) and M0 (one-ratio) vs. M3 (discrete) were calculated in order to detect positive selection [79].

### 4.6. Phylogenetic Analysis

In order to deeply detect the evolutionary relationship of the Orchidaceae family, 50 available complete chloroplast genomes were downloaded from the NCBI Organelle Genome Resources database (Table S1). In addition, *Artemisia argyi* and *Megadenia pygmaea* were used as outgroups. In total, 54 nucleotide sequences of complete chloroplast genomes were aligned using MAFFT [73]; the detailed parameters were as follows: 200 PAM/K = 2 and 1.53 gap open penalty [73]. The choice of the best nucleotide sequence substitution model (GTRGAMMA model) was determined using the Modeltest v3.7 [80]. We constructed a maximum likelihood phylogenetic tree based on these complete plastomes using MAGA7 [34] with 1000 bootstrap replicates under the GTRGAMMA model [80].

## Figures and Tables

**Table 1:** Comparison of chloroplast genome features in four orchid species.

Species	*Cremastra appendiculata*	*Calanthe davidii*	*Epipactis mairei*	*Platanthera japonica*
Accession number	MG925366	MG925365	MG925367	MG925368
Genome size (bp)	155,320	153,629	160,427	154,995
LSC length (bp)	87,098	86,045	88,328	85,979
SSC length (bp)	15,478	15,672	18,513	13,664
IR length (bp)	26,372	25,956	26,790	27,676
Coding (bp)	100,018	104,531	113,915	107,028
Non-coding (bp)	55,302	49,098	46,512	47,967
Number of genes	130 (0)	132 (19)	131 (19)	128 (17)
Number of protein-coding genes	83 (7)	86 (7)	85 (7)	85 (7)
Number of tRNA genes	38 (8)	38 (8)	38 (8)	38 (8)
Number of rRNA genes	8 (4)	8 (4)	8 (4)	8 (4)
GC content (%)	37.2	36.9	37.2	37
GC content in LSC (%)	34.5	34.5	34.9	34.2
GC content in SSC (%)	30.4	30.2	31.0	29
GC content in IR (%)	43.5	43.1	43.1	43.2
Mapped read number	551,680	324,741	230,968	322,259
Chloroplast coverage	544.9	217.4	216	313.6

The numbers in parenthesis indicate the genes duplicated in the IR regions.

**Table 2:** List of genes present in four orchid chloroplast genomes.

Category of Genes	Group of Gene	Name of Gene	Name of Gene	Name of Gene	Name of Gene	Name of Gene
Self-replication	Ribosomal RNA genes	*rrn16* ^(×2)^	*rrn2* ^(×2)^	*rrn4.5* ^(×2)^	*rrn5* ^(×2)^	
	Transfer RNA genes	*trnA*-*UGC* *^,(×2)^	*trnC*-*GCA*	*trnD*-*GUC*	*trnE*-*UUC*	*trnF*-*GAA*
		*trnfM*-*CAU*	*trnG*-*GCC* *	*trnG*-*UCC*	*trnH*-*GUG* ^(×2)^	*trnI*-*CAU* ^(×2)^
		*trnI*-*GAU* *^,(×2)^	*trnK*-*UUU* *	*trnL*-*CAA* ^(×2)^	*trnL*-*UAA* *	*trnL*-*UAG*
		*trnM*-*CAU*	*trnN*-*GUU* ^(×2)^	*trnP*-*UGG*	*trnQ*-*UUG*	*trnR*-*ACG* ^(×2)^
		*trnR*-*UCU*	*trnS*-*GCU*	*trnS*-*GGA*	*trnS*-*UGA*	*trnT*-*GGU*
		*trnT*-*UGU*	*trnV*-*GAC* ^(×2)^	*trnV*-*UAC* ^(×2)^	*trnW*-*CCA*	*trnY*-*GUA*
	Small subunit of ribosome	*rps2*	*rps3*	*rps4*	*rps7* ^(×2)^	*rps8*
		*rps11*	*rps12* **^,(×2)^	*rps14*	*rps15*	*rps16* *
		*rps18*	*rps19* ^(×2)^			
	Large subunit of ribosome	*rpl2* *^,(×2)^	*rpl14*	*rpl16* *	*rpl20*	*rpl22*
		*rpl23* ^(×2)^	*rpl32*	*rpl33*	*rpl36*	
	DNA-dependent RNA polymerase	*rpoA*	*rpoB*	*rpoC1* *	*rpoC2*	
	Translational initiation factor	*infA*				
Genes for photosynthesis	Subunits of NADH-dehydrogenase	*ndhA* *	*ndhB* *^,(×2)^	*ndhC* ^a^	*ndhD*	*ndhE*
		*ndhF*	*ndhG*	*ndhH*	*ndhI* ^a,c,d^	*ndhJ*
		*ndhK* ^a^				
	Subunits of photosystem I	*psaA*	*psaB*	*psaC*	*psaI*	*psaJ*
		*ycf3* **	*ycf4*			
	Subunits of photosystem II	*psbA*	*psbB*	*psbC*	*psbD*	*psbE*
		*psbF*	*psbH*	*psbI*	*psbJ*	*psbK*
		*psbL*	*psbM*	*psbN*	*psbT*	*psZ*
	Subunits of cytochrome b/f complex	*petA*	petB*	petD *	*petG*	*petL*
		*petN*				
	Subunits of ATP synthase	*atp A*	*atp B*	*atp E*	*atp F* *	*atp H*
		*atpI*				
	Subunits of rubisco	*rbcL*				
Other genes	Maturase	*matK*				
	Protease	*clpP* **				
	Envelope membrane protein	*cemA*				
	Subunit of acetyl-CoA carboxylase	*accD*				
	C-type cytochrome synthesis gene	*ccsA*				
Genes of unknown function	Conserved open reading frames	*ycf1*	*ycf2* ^(×2)^			

^a^ gene is no in *Cremastra appendiculata*; ^c^ gene is not in *Epipactis mairei*; ^d^ gene is not in *Platanthera japonica*; * Gene contains one intron; ** gene contains two introns; ^(×2)^ indicates that the number of the repeat unit is 2.

**Table 3:** List of taxa sampled in the study and species accessions numbers (GenBank).

Subfamily	Species	Accession Number
Orchidaceae subfamily. Epidendroideae	*Cattleya crispata*	KP168671
	*Cremastra appendiculata*	MG925366
	*Masdevallia coccinea*	KP205432
	*Erycina pusilla*	JF746994
	*Phalaenopsis equestris*	JF719062
	*Bletilla ochracea*	KT695602
	*Cymbidium faberi*	KR919606
	*Calanthe davidii*	MG925365
	*Dendrobium strongylanthum*	KR673323
	*Elleanthus sodiroi*	KR260986
	*Sobralia callosa*	KM032623
Orchidaceae subfamily. Orchidoideae	*Epipactis mairei*	MG925367
	*Cephalanthera longifolia*	KU551263
	*Listera fugongensis*	KU551270
	*Platanthera japonica*	MG925368
	*Habenaria pantlingiana*	KJ524104
	*Goodyera velutina*	KT886432
	*Anoectochilus emeiensis*	LC057212
	*Ludisia discolor*	KU578274
Orchidaceae subfamily. Vanilloideae	*Vanilla planifolia*	KJ566306

## Supplementary Materials

The following are available online at http://www.mdpi.com/1422-0067/19/3/716/s1.
