# Supplementary material for: Molecular Evolution of Chloroplast Genomes of Orchid Species: Insights into Phylogenetic Relationship and Adaptive Evolution
Source: Int J Mol Sci. 2018 Mar 2;19(3):716. doi: 10.3390/ijms19030716 (PMC5877577; doi:10.3390/ijms19030716)
Supplement: Supplementary file 1 [file ijms-19-00716-s001.pdf]

Table S1. List of taxa sampled in this study and species accessions numbers (GenBank).

Subfamily	Species	Accession number
Orchidaceae subfam. Epidendroideae	*Cymbidium aloifolium*	KC876122
	Cymbidium ensifolium	KU179434
	Cymbidium faberi	KR919606
	Cymbidium goeringii	KT722982
	Cymbidium kanran	KU179435
	Cymbidium lancifolium	KU179436
	Cymbidium macrorhizon	KU179437
	Cymbidium mannii	KC876126
	Cymbidium sinense	KC876123
	Cymbidium tortisepalum	KC876124
	Cymbidium tracyanum	KC876127
	Dendrobium huoshanense	KT630834
	Dendrobium nobile	KX377961
	Dendrobium officinale	KC771275
	Dendrobium pendulum	KT695604
	Dendrobium strongylanthum	KR673323
	Bletilla ochracea	KT695602
	Bletilla striata	KT588924
	Cattleya crispata	KP168671
	Cattleya liliputana	NC_032083
	Masdevallia coccinea	KP205432
	Masdevallia picturata	NC_026777
	Erycina pusilla	JF746994
	Oncidium Gower Ramsey	GQ324949
	Phalaenopsis aphrodite subsp. Formosana	AY916449
	Phalaenopsis equestris	JF719062
	Calanthe triplicata	KF753635
	Calanthe davidii	MG925365
	Cremastra appendiculata	MG925366
	Sobralia callosa	KM032623
	Elleanthus sodiroi	KR260986
	Corallorhiza striata var. vreelandii	JX087681
Orchidaceae subfam. Orchidoideae	*Habenaria pantlingiana*	KJ524104
	Goodyera fumata	KJ501999
	Goodyera procera	KT886429
	Goodyera schlechtendaliana	KT886431

	Goodyera velutina	KT886432
	Epipactis mairei	MG925367
	Epipactis veratrifolia	KU551267
	Anoectochilus emeiensis	LC057212
	Cephalanthera longifolia	KU551263
	Ludisia discolor	KU578274
	Listera fugongensis	KU551270
	Platanthera japonica	MG926368
Orchidaceae subfam. Vanilloideae	*Vanilla planifolia*	KJ566306
Orchidaceae subfam. Cypripedioideae	*Paphiopedilum armeniacum*	KJ566307
	Paphiopedilum niveum	KJ524105
	Phragmipedium longifolium	KM032625
Orchidaceae subfam. Apostasioideae	*Apostasia odorata*	KM244734
	Neuwiedia singapureana	KM244735
Outgroup	*Artemisia argyi*	KM386991
	Megadenia pygmaea	NC_034357

Table S2. The repeats distribution in the chloroplast genomes of four Orchidaceae species.

Table S2a. The repeats distribution in *Cremastra appendiculata* chloroplast genome.

	Length of the 1st repeat	Starting position of the 1st repeat	Direction	Length of the 2nd repeat	Starting position of the 2nd repeat	Distance between the repeats	Evalue	RegionA	RegionB
Dispersed repeats	203	30108	F	203	30311	0	4.11E-113	LSC;IGS(petN-psbM)	LSC;IGS(petN-psbM)
	150	50518	F	150	50670	0	3.33E-81	LSC;IGS(trnF-GAA-ndhJ)	LSC;IGS(trnF-GAA-ndhJ)
	148	29384	F	148	29530	0	5.33E-80	LSC;IGS(trnC-GCA-petN)	LSC;IGS(trnC-GCA-petN)
	141	89163	F	141	89541	0	8.73E-76	IRB;IGS(rpl23-rpl2)	IRB;CDS(rpl23)
	141	152736	F	141	153114	0	8.73E-76	IRA;CDS(rpl23)	IRA;IGS(rpl23-rpl2)
	128	48078	F	128	48205	0	5.86E-68	LSC;IGS(trnT-UGU-trnL-UAA)	LSC;IGS(trnT-UGU-trnL-UAA)
	123	89066	F	123	89419	-2	4.05E-60	IRB;IGS(rpl2)	IRB;IGS(rpl2-rpl23)
	123	152876	F	123	153229	-2	4.05E-60	IRA;IGS(rpl23-rpl2)	IRA;IGS(rpl2)
	113	125502	F	113	125614	0	6.29E-59	SSC;IGS(rps15-ycf1)	SSC;CDS(ycf1)
	109	7837	F	109	7944	0	1.61E-56	LSC;IGS(trnQ-UUG-psbK)	LSC;IGS(trnQ-UUG-psbK)
	102	122548	F	102	122647	0	2.64E-52	SSC;IGS(ndhH-rps15)	SSC;IGS(ndhH-rps15)
	100	64572	F	100	64681	0	4.22E-51	LSC;IGS(petA-psbJ)	LSC;IGS(petA-psbJ)
	99	103492	F	99	103590	0	1.69E-50	IRB;IGS(rps12-trnV-GAC)	IRB;IGS(rps12-trnV-GAC)
	99	138729	F	99	138827	0	1.69E-50	IRA;IGS(trnV-GAC-rps12)	IRA;IGS(trnV-GAC-rps12)
	98	44683	F	98	44780	0	6.76E-50	LSC;CDS(ycf3 intron)	LSC;CDS(ycf3 intron)
	96	67043	F	96	67142	0	1.08E-48	LSC;IGS(psbE-petL)	LSC;IGS(psbE-petL)
	86	50244	F	86	50344	0	1.13E-42	LSC;IGS(trnF-GAA-ndhJ)	LSC;IGS(trnF-GAA-ndhJ)
	86	124268	F	86	124366	0	1.13E-42	LSC;IGS(rps15-ycf1)	LSC;IGS(rps15-ycf1)
	84	3824	F	84	3914	0	1.81E-41	LSC;tRNA(trnK-UUU intron)	LSC;tRNA(trnK-UUU intron)
	94	0	F	94	155226	-3	6.26E-41	LSC;IGS(rps19-psbA)	IRA;CDS(rps19)
	85	115744	F	85	115837	-1	1.16E-39	SSC;IGS(rpl32-trnL-UAG)	SSC;IGS(rpl32-trnL-UAG)
	81	37465	F	81	37560	0	1.16E-39	LSC;IGS(lhbA-trnG-GCC)	LSC;IGS(lhbA-trnG-GCC)
	73	71222	F	73	71307	0	7.61E-35	LSC;IGS(rpl20-clpP)	LSC;IGS(rpl20-clpP)
	59	46732	F	59	46801	0	2.04E-26	LSC;IGS(trnS-GGA-rps4)	LSC;IGS(trnS-GGA-rps4)
	55	120276	F	55	120344	-3	1.05E-25	SSC;IGS(ndhE-ndhG)	SSC;IGS(ndhE-ndhG)
	56	34099	F	56	34177	0	1.31E-24	LSC;IGS(trnT-GGU-psbD)	LSC;IGS(trnT-GGU-psbD)
	54	33434	F	54	33504	0	2.09E-23	LSC;IGS(trnT-GGU-psbD)	LSC;IGS(trnT-GGU-psbD)
	50	44	F	50	155270	0	5.35E-21	LSC;IGS(rps19-psbA)	LSC;IGS(rps19-psbA)
	47	8941	F	47	9008	0	3.43E-19	LSC;IGS(psbI-trnS-GCU)	LSC;IGS(psbI-trnS-GCU)

	43	70788	F	43	70842	0	8.77E-17	LSC;IGS(rpl20-clpP)	LSC;IGS(rpl20-clpP)
	45	37851	F	45	37913	-1	7.40E-16	LSC;IGS(trnG-GCC-trnfM-CAU)	LSC;IGS(trnG-GCC-trnfM-CAU)
	41	23674	F	41	23728	0	1.40E-15	LSC;CDS(rpoC1 intron)	LSC;CDS(rpoC1 intron)
	37	8623	F	37	8676	0	3.59E-13	LSC;IGS(psbK-psbI)	LSC;IGS(psbK-psbI)
	34	89331	F	34	89656	0	2.30E-11	IRB;IGS(rpl2-rpl23)	IRB;CDS(rpl23)
	34	123340	F	34	123398	0	2.30E-11	SSC;IGS(rps15-ycf1)	SSC;IGS(rps15-ycf1)
	34	152728	F	34	153053	0	2.30E-11	IRA;CDS(rpl23)	IRA;IGS(rpl23-rpl2)
	43	55451	F	43	55505	-3	2.92E-11	LSC;IGS(atpB-rbcL)	LSC;IGS(atpB-rbcL)
	32	6160	F	32	6221	0	3.68E-10	LSC;IGS(rps16-trnQ-UUG)	LSC;IGS(rps16-trnQ-UUG)
	30	116262	F	30	116327	0	5.89E-09	SSC;IGS(rpl32-trnL-UAG)	SSC;IGS(rpl32-trnL-UAG)
	35	9850	F	35	9893	-3	1.02E-06	LSC;IGS(trnS-GCU-trnG-UCC)	LSC;IGS(trnS-GCU-trnG-UCC)
	30	40072	F	30	42296	-2	2.30E-05	LSC;CDS(psaB)	LSC;IGS(psaA)
	30	89305	F	30	89327	-2	2.30E-05	IRB;IGS(rpl2-rpl23)	IRB;IGS(rpl2-rpl23)
	30	153061	F	30	153083	-2	2.30E-05	IRA;IGS(rpl23-rpl2)	IRA;IGS(rpl23-rpl2)
	39	4233	R	39	4233	0	2.24E-14	LSC;tRNA(trnK-UUU intron)	LSC;tRNA(trnK-UUU intron)
	30	123176	R	30	123176	-2	2.30E-05	SSC;IGS(RPS15-ycf1)	SSC;IGS(RPS15-ycf1)
Palindromic repeats	141	89163	P	141	152736	0	8.73E-76	IRB;IGS(rpl23-rpl2)	IRA;CDS(rpl23)
	141	89541	P	141	153114	0	8.73E-76	IRB;CDS(rpl23)	IRA;IGS(rpl23-rpl2)
	139	251	P	139	86767	0	1.40E-74	LSC;IGS(rps19-psbA)	LSC;CDS(rpl22)
	123	89066	P	123	152876	-2	4.05E-60	IRB;IGS(rpl2)	IRA;IGS(rpl23-rpl2)
	123	89419	P	123	153229	-2	4.05E-60	IRB;IGS(rpl2-rpl23)	IRA;IGS(rpl2)
	99	103492	P	99	138729	0	1.69E-50	IRB;IGS(rps12-trnV-GAC)	IRA;IGS(trnV-GAC-rps12)
	99	103590	P	99	138827	0	1.69E-50	IRB;IGS(rps12-trnV-GAC)	IRA;IGS(trnV-GAC-rps12)
	31	0	P	31	87162	-3	4.17E-42	LSC;IGS(rps19-psbA)	IRB;CDS(rps19)
	50	44	P	50	87098	-3	3.35E-22	LSC;IGS(rps19-psbA)	IRB;CDS(rps19)
	30	439	P	30	470	-1	2.34E-25	LSC;IGS(rps19-psbA)	LSC;IGS(rps19-psbA)
	48	5693	P	48	5693	0	8.56E-20	LSC;IGS(rps16-trnQ-UUG)	LSC;IGS(rps16-trnQ-UUG)
	46	30707	P	46	30707	0	1.37E-18	LSC;IGS(petN-psbM)	LSC;IGS(petN-psbM)
	45	64610	P	45	64719	-1	7.40E-16	LSC;IGS(petA-psbJ)	LSC;IGS(petA-psbJ)
	31	113	P	31	87040	-2	8.69E-16	LSC;IGS(rps19-psbA)	LSC;IGS(rpl22-rps19)
	47	65361	P	47	65361	-3	1.50E-13	LSC;IGS(petA-psbJ)	LSC;IGS(petA-psbJ)
	36	37918	P	36	37918	0	1.44E-12	LSC;IGS(trnG-GCC-trnfM-CAU)	LSC;IGS(trnG-GCC-trnfM-CAU)
	36	83453	P	36	83453	0	1.44E-12	LSC;CDS(rpl14)	LSC;CDS(rpl14)

	35	37856	P	35	37919	0	5.75E-12	LSC;IGS(trnG-GCC-trnfM-CAU)	LSC;IGS(trnG-GCC-trnfM-CAU)
	34	37857	P	34	37857	0	2.30E-11	LSC;IGS(trnG-GCC-trnfM-CAU)	LSC;IGS(trnG-GCC-trnfM-CAU)
	34	89331	P	34	152728	0	2.30E-11	IRB;IGS(rpl2-rpl23)	IRA;CDS(rpl23)
	34	89656	P	34	153053	0	2.30E-11	IRB;CDS(rpl23)	IRA;IGS(rpl23-rpl2)
	39	45133	P	39	139830	-3	5.54E-09	LSC;CDS(ycf3 intron)	IRA;IGS(trnV-GAC-rps12)
	39	123152	P	39	123152	-3	5.54E-09	SSC;IGS(rps15-ycf1)	SSC;IGS(rps15-ycf1)
	30	48110	P	30	48237	0	5.89E-09	LSC;IGS(trnT-UGU-trnL-UAA)	LSC;IGS(trnT-UGU-trnL-UAA)
	30	117117	P	30	117117	0	5.89E-09	SSC;CDS(ccsA)	SSC;CDS(ccsA)
	32	200	P	32	86943	-1	3.53E-08	LSC;IGS(rps19-psbA)	LSC;IGS(rpl22-rps19)
	37	124623	P	37	124623	-3	7.54E-08	SSC;IGS(rps15-ycf1)	SSC;IGS(rps15-ycf1)
	31	9107	P	31	46566	-1	1.37E-07	LSC;tRNA(trnS-GCU)	LSC;tRNA(trnS-GGA)
	35	85725	P	35	85725	-3	1.02E-06	LSC;IGS(rpl16-rps3)	LSC;IGS(rpl16-rps3)
	35	116546	P	35	116546	-3	1.02E-06	SSC;IGS(trnL-UAG-ccsA)	SSC;IGS(trnL-UAG-ccsA)
	30	120730	P	30	120730	-2	1.64E-06	SSC;IGS(ndhG-ndhA)	SSC;IGS(ndhG-ndhA)
	30	89305	P	30	153061	-2	2.30E-05	IRB;IGS(rpl2-rpl23)	IRA;IGS(rpl23-rpl2)
	30	89327	P	30	153083	-2	2.30E-05	IRB;IGS(rpl2-rpl23)	IRA;IGS(rpl23-rpl2)

Table S2b The repeats distribution in *Calanthe davidii* chloroplast genome.

	Length of the 1st repeat	Starting position of the 1st repeat	Direction	Length of the 2nd repeat	Starting position of the 2nd repeat	Distance between the repeats	Evalue	RegionA	RegionB
Dispersed repeats	150	49319	F	150	49575	0	3.26E-81	LSC; IGS, tRNA (trnL-UAA-trnF-GAA, trnF-GAA)	LSC; tRNA, IGS (trnF-GAA, trnF-GAA-ndhJ)
	137	84404	F	137	101709	0	2.19E-73	LSC; CDS (rpl16 intron)	IRb; IGS (rps12-trnV-GAC)
	117	54835	F	117	54951	0	2.40E-61	LSC; IGS (atpB-rbcL)	LSC; IGS (atpB-rbcL)
	100	45482	F	100	45593	0	4.13E-51	LSC; IGS (ycf3-trnS-GGA)	LSC; IGS (ycf3-trnS-GGA)
	97	114518	F	97	114611	0	2.64E-49	SSC; IGS (rpl32-trnL-UAG)	SSC; IGS (rpl32-trnL-UAG)
	83	42717	F	83	42813	0	7.10E-41	LSC; IGS (psaA-ycf3)	LSC; IGS (psaA-ycf3)
	82	50950	F	82	51044	0	2.84E-40	LSC; CDS (ndhK)	LSC; CDS, CDS (ndhK, ndhC)
	79	62496	F	79	62591	0	1.82E-38	LSC; CDS, IGS (cemA, cemA-petA)	LSC; IGS (cemA-petA)
	74	111166	F	74	111238	0	1.86E-35	IRb; IGS (trnR-ACG-trnN-GUU)	IRb; IGS (trnR-ACG-trnN-GUU)
	74	128362	F	74	128434	0	1.86E-35	IRa; IGS (trnN-GUU-trnR-ACG)	IRa; IGS (trnN-GUU-trnR-ACG)
	69	83770	F	69	83845	-1	3.94E-30	LSC; CDS (rpl16 intron)	LSC; CDS (rpl16 intron)

	63	84	F	63	153566	0	7.80E-29	LSC; IGS (rps19-psbA)	IRa; IGS (rps19-psbA)
	65	47574	F	65	47653	-1	9.51E-28	LSC; IGS (trnT-UGU-trnL-UAA)	LSC; IGS (trnT-UGU-trnL-UAA)
	58	14051	F	58	14110	0	7.99E-26	LSC;IGS (atpF-atpH)	LSC;IGS, CDS (atpF-atpH, atpH)
	56	114959	F	56	115017	0	1.28E-24	SSC; IGS (rpl32-trnL-UAG)	SSC; IGS (rpl32-trnL-UAG)
	56	123664	F	56	123727	0	1.28E-24	SSC; CDS (ycf1)	SSC; CDS (ycf1)
	56	33658	F	56	33736	-1	2.15E-22	LSC; IGS (trnT-GGU-psbD)	LSC; IGS (trnT-GGU-psbD)
	52	91115	F	52	91167	0	3.27E-22	IRb; IGS, CDS (trnI-CAU-ycf2, ycf2)	IRb; CDS (ycf2)
	52	148455	F	52	148507	0	3.27E-22	IRa; CDS (ycf2)	IRa; CDS, IGS (ycf2, trnI-CAU-ycf2)
	49	37028	F	49	37108	0	2.09E-20	LSC; IGS (psbZ-trnG-UCC)	LSC; IGS (psbZ-trnG-UCC)
	47	86180	F	47	86219	-1	4.73E-17	IRb; IGS (rpl22-rps19)	IRb; IGS (rpl22-rps19)
	47	153408	F	47	153447	-1	4.73E-17	IRa; IGS (rps19-rpl22)	IRa; IGS (rps19-rpl22)
	39	44585	F	39	101362	-2	1.46E-10	LSC; CDS (ycf3 intron2)	IRb; IGS (rps12-trnV-GAC)
	39	44585	F	39	101903	-2	1.46E-10	LSC; CDS (ycf3 intron2)	IRb; IGS (rps12-trnV-GAC)
	31	93957	F	31	93975	0	1.44E-09	IRb; CDS (ycf2)	IRb; CDS (ycf2)
	31	145668	F	31	145686	0	1.44E-09	IRa; CDS (ycf2)	IRa; CDS (ycf2)
	30	73689	F	30	73739	-2	2.25E-05	LSC; IGS (clpP-psbB)	LSC; IGS (clpP-psbB)
	31	14986	F	31	15008	-3	1.75E-04	LSC; IGS (atpH-atpI)	LSC; IGS (atpH-atpI)
	30	39587	F	30	41811	-3	6.31E-04	LSC; CDS (psaB)	LSC; CDS (psaA)
	30	46282	F	30	46295	-3	6.31E-04	LSC; IGS (trnS-GGA-rps4)	LSC; IGS (trnS-GGA-rps4)
Palindromic repeats	63	84	P	63	86045	0	7.80E-29	LSC; IGS (rps19-psbA)	IRb; CDS, IGS (rpl22, rpl22-rps19)
	59	167	P	59	167	-1	3.54E-24	LSC; IGS (rps19-psbA)	LSC; IGS (rps19-psbA)
	52	91115	P	52	148455	0	3.27E-22	IRb; IGS, CDS (trnI-CAU-ycf2, ycf2)	IRa; CDS (ycf2)
	52	91167	P	52	148507	0	3.27E-22	IRb; CDS (ycf2)	IRa; CDS, IGS (ycf2, trnI-CAU-ycf2)
	48	30228	P	48	30228	0	8.38E-20	LSC; IGS (petN-psbM)	LSC; IGS (petN-psbM)
	57	64013	P	57	64013	-3	2.52E-19	LSC; IGS (petA-psbJ)	LSC; IGS (petA-psbJ)
	47	86180	P	47	153408	-1	4.73E-17	IRb; IGS (rpl22-rps19)	IRa; IGS (rps19-rpl22)
	47	86219	P	47	153447	-1	4.73E-17	IRb; IGS (rpl22-rps19)	IRa; IGS (rps19-rpl22)

	34	119435	P	34	119435	0	2.25E-11	SSC; IGS (ndhI-ndhA)	SSC; IGS (ndhI-ndhA)
	39	44585	P	39	137732	-2	1.46E-10	LSC; CDS (ycf3 intron2)	IRa; IGS (trnV-GAC-rps12)
	39	44585	P	39	138273	-2	1.46E-10	LSC; CDS (ycf3 intron2)	IRa; IGS (trnV-GAC-rps12)
	32	28273	P	32	28273	0	3.60E-10	LSC; IGS (rpoB-trnC-GCA)	LSC; IGS (rpoB-trnC-GCA)
	31	93957	P	31	145668	0	1.44E-09	IRb; CDS (ycf2)	IRa; CDS (ycf2)
	31	93975	P	31	145686	0	1.44E-09	IRb; CDS (ycf2)	IRa; CDS (ycf2)
	36	29295	P	36	29295	-2	7.97E-09	LSC; IGS (trnC-GCA-petN)	LSC; IGS (trnC-GCA-petN)
	33	29690	P	33	29690	-1	8.91E-09	LSC; IGS (petN-psbM)	LSC; IGS (petN-psbM)
	37	121800	P	37	121800	-3	7.37E-08	SSC; IGS (rps15-ycf1)	SSC; IGS (rps15-ycf1)
	37	123170	P	37	123170	-3	7.37E-08	SSC; CDS (ycf1)	SSC; CDS (ycf1)
	30	8619	P	30	46049	-1	5.18E-07	LSC; tRNA (trnS-GCU)	LSC; tRNA (trnS-GGA)
	31	54839	P	31	118271	-2	6.02E-06	LSC; IGS (atpB-rbcL)	SSC; IGS (psaC-ndhE)
	31	54955	P	31	118271	-2	6.02E-06	LSC; IGS (atpB-rbcL)	SSC; IGS (psaC-ndhE)
	30	37411	P	30	37411	-2	2.25E-05	LSC; IGS (trnG-UCC-trnfM-CAU)	LSC; IGS (trnG-UCC-trnfM-CAU)
	32	36496	P	32	46049	-3	4.82E-05	LSC; IGS, tRNA (psbC-trnS-UGA, trnS-UGA)	LSC; tRNA, CDS (trnS-GGA, trnS-GGA-rps4)
	32	49993	P	32	50077	-3	4.82E-05	LSC; IGS (trnF-GAA-ndhJ)	LSC; IGS, CDS (trnF-GAA-ndhJ, ndhJ)
	31	1793	P	31	1793	-3	1.75E-04	LSC, tRNA (trnK-UUU intron)	LSC, tRNA (trnK-UUU intron)
	30	44597	P	30	137729	-3	6.31E-04	LSC; CDS (ycf3 intron2)	IRa; IGS (trnV-GAC-rps12)
	30	44597	P	30	138270	-3	6.31E-04	LSC; CDS (ycf3 intron2)	IRa; IGS (trnV-GAC-rps12)

Table S2c The repeats distribution in *Epipactis mairei* chloroplast genome.

	Length of the 1st repeat	Starting position of the 1st repeat	Direction	Length of the 2nd repeat	Starting position of the 2nd repeat	Distance between the repeats	Evalue	RegionA	RegionB
Dispersed repeats	309	8951	F	309	9258	0	6.11E-177	LSC; IGS (trnS-GCU-trnG-GCC)	LSC; IGS (trnS-GCU-trnG-GCC)
	192	3430	F	192	3620	0	1.69E-106	LSC; tRNA (trnK-UUU intron)	LSC; tRNA (trnK-UUU intron)
	179	59080	F	179	59261	0	1.13E-98	LSC; IGS (accD-psaI)	LSC; IGS (accD-psaI)
	166	2	F	166	153523	0	7.59E-91	LSC; IGS (rpl22-psbA)	IRa; IGS (rps19-rpl22)
	158	48351	F	158	48508	0	4.98E-86	LSC; tRNA (trnL-UAA intron)	LSC; tRNA (trnL-UAA intron)

155	13139	F	155	13294	0	3.18E-84	LSC; CDS (atpF intron)	LSC; CDS (atpF intron)
149	28201	F	149	28352	0	1.30E-80	LSC; IGS (rpoB-trnC-GCA)	LSC; IGS (rpoB-trnC-GCA)
144	75474	F	144	75610	0	1.34E-77	LSC; IGS (psbB-psbT)	LSC; IGS (psbB-psbT)
130	32313	F	130	32441	0	3.59E-69	LSC; IGS (trnE-UUC-trnT-GGU)	LSC; IGS (trnE-UUC-trnT-GGU)
129	49460	F	129	49861	0	1.43E-68	LSC; IGS (trnF-GAA-ndhJ)	LSC; IGS (trnF-GAA-ndhJ)
110	122646	F	110	122768	0	3.94E-57	SSC; CDS (ycf1)	SSC; CDS (ycf1)
116	7909	F	116	8019	-2	5.78E-56	LSC; IGS (psbK-psbI)	LSC; IGS (psbK-psbI)
118	68124	F	118	68228	-3	4.34E-55	LSC; IGS (psbJ-rpl33)	LSC; IGS (psbJ-rpl33)
102	99897	F	102	99997	0	2.58E-52	IRb; IGS (ndhB-rps7)	IRb; IGS (ndhB-rps7)
102	139665	F	102	139765	0	2.58E-52	IRa; IGS (rps7-ndhB)	IRa; IGS (rps7-ndhB)
82	125536	F	82	125617	-1	6.99E-38	SSC; CDS (ycf1)	SSC; CDS (ycf1)
75	49031	F	75	49171	0	4.65E-36	LSC; IGS (trnL-UAA-trnF-GAA)	LSC; IGS (trnL-UAA-trnF-GAA)
72	43564	F	72	43652	0	2.98E-34	LSC; CDS (ycf3 intron1)	LSC; CDS (ycf3 intron1)
70	48975	F	70	49104	0	4.77E-33	LSC; IGS (trnL-UAA-trnF-GAA)	LSC; IGS (trnL-UAA-trnF-GAA)
70	65774	F	70	65864	0	4.77E-33	LSC; IGS (psbE-petL)	LSC; IGS (psbE-petL)
71	6693	F	71	6764	-1	2.54E-31	LSC; IGS (rps16-trnQ-UUG)	LSC; IGS (rps16-trnQ-UUG)
63	14359	F	63	14441	0	7.81E-29	LSC; IGS (atpH-atpI)	LSC; IGS (atpH-atpI)
66	16097	F	66	16177	-1	2.42E-28	LSC; IGS (atpI-rps2)	LSC; IGS (atpI-rps2)
61	56825	F	61	56899	0	1.25E-27	LSC; IGS (rbcL-accD)	LSC; IGS (rbcL-accD)
62	102397	F	62	102451	-1	5.81E-26	IRb; IGS (rps12-trnV-GAC)	IRb; IGS (rps12-trnV-GAC)
62	137251	F	62	137305	-1	5.81E-26	IRa; IGS (trnV-GAC -rps12)	IRa; IGS (trnV-GAC -rps12)
54	64166	F	54	64243	0	2.05E-23	LSC; IGS (petA-psbJ)	LSC; IGS (petA-psbJ)
47	13992	F	47	14052	0	3.35E-19	LSC; IGS (atpF-atpH)	LSC; IGS (atpF-atpH)
47	95901	F	47	95948	0	3.35E-19	IRb; IGS (ycf2-trnL-CAA)	IRb; IGS (ycf2-trnL-CAA)
47	143769	F	47	143816	0	3.35E-19	IRa; IGS (trnL-CAA-ycf2)	IRa; IGS (trnL-CAA-ycf2)
46	29845	F	46	29908	0	1.34E-18	LSC; IGS (petN-psbM)	LSC; IGS (petN-psbM)
45	33559	F	45	33627	0	5.37E-18	LSC; IGS (trnT-GGU-psbG)	LSC; IGS (trnT-GGU-psbG)
49	91558	F	49	91579	-2	2.22E-16	IRb; CDS (ycf2)	IRb; CDS (ycf2)
49	148136	F	49	148157	-2	2.22E-16	IRa; CDS (ycf2)	IRa; CDS (ycf2)
42	54712	F	42	54779	0	3.43E-16	LSC; IGS (atpB-rbcL)	LSC; IGS (atpB-rbcL)
40	61528	F	40	61575	-1	6.59E-13	LSC; IGS (ycf4-cemA)	LSC; IGS (ycf4-cemA)
36	78942	F	36	78991	0	1.41E-12	LSC; CDS (petD intron)	LSC; CDS (petD intron)

	36	94001	F	36	94019	0	1.41E-12	IRb; CDS (ycf2)	IRb; CDS (ycf2)
	36	145709	F	36	145727	0	1.41E-12	IRa; CDS (ycf2)	IRa; CDS (ycf2)
	36	111333	F	36	111367	0	1.41E-12	IRb; IGS (trnR-ACG- trnN-GUU)	IRb; IGS (trnR-ACG- trnN-GUU)
	36	128361	F	36	128395	0	1.41E-12	IRa; IGS (trnN-GUU-trnR-ACG)	IRa; IGS (trnN-GUU-trnR-ACG)
	35	111649	F	35	111689	0	5.63E-12	IRb; IGS (trnR-ACG- trnN-GUU)	IRb; IGS (trnR-ACG- trnN-GUU)
	35	128040	F	35	128080	0	5.63E-12	IRa; IGS (trnN-GUU-trnR-ACG)	IRa; IGS (trnN-GUU-trnR-ACG)
	34	115158	F	34	115220	0	2.25E-11	SSC; IGS (trnL-UAG-ccsA)	SSC; IGS (trnL-UAG-ccsA)
	32	121183	F	32	121235	0	3.60E-10	SSC; IGS (ndhH-rps15)	SSC; IGS (ndhH-rps15)
	31	114547	F	31	114595	0	1.44E-09	LSC; IGS (rpl32-trnL-UAG)	LSC; IGS (rpl32-trnL-UAG)
	33	75450	R	33	75450	0	9.00E-11	LSC; IGS (psbB-psbT)	LSC; IGS (psbB-psbT)
	33	73331	R	33	73335	-3	1.33E-05	LSC; IGS (clpP-psbB)	LSC; IGS (clpP-psbB)
	32	9018	R	32	9316	-3	4.82E-05	LSC; IGS (trnS-GCU-trnG-GCC)	LSC; IGS (trnS-GCU-trnG-GCC)
	32	75450	C	32	75451	0	3.60E-10	LSC; IGS (psbB-psbT)	LSC; IGS (psbB-psbT)
Palindromic repeats	167	2	P	167	86074	0	1.90E-91	LSC; IGS (rpl22-psbA)	IRb; CDS, IGS (rpl22, rpl22-rps19)
	102	99897	P	102	139665	0	2.58E-52	IRb; IGS (ndhB-rps7)	IRa; IGS (rps7-ndhB)
	102	99997	P	102	139765	0	2.58E-52	IRb; IGS (ndhB-rps7)	IRa; IGS (rps7-ndhB)
	62	102397	P	62	137251	-1	5.81E-26	IRb; IGS (rps12-trnV-GAC)	IRa; IGS (trnV-GAC -rps12)
	62	102451	P	62	137305	-1	5.81E-26	IRb; IGS (rps12-trnV-GAC)	IRa; IGS (trnV-GAC -rps12)
	47	95901	P	47	143769	0	3.35E-19	IRb; IGS (ycf2-trnL-CAA)	IRa; IGS (trnL-CAA-ycf2)
	47	95948	P	47	143816	0	3.35E-19	IRb; IGS (ycf2-trnL-CAA)	IRa; IGS (trnL-CAA-ycf2)
	46	30355	P	46	30355	0	1.34E-18	LSC; IGS (petN-psbM)	LSC; IGS (petN-psbM)
	52	170	P	52	170	-2	3.91E-18	LSC; IGS (rpl22-psbA)	LSC; IGS (rpl22-psbA)
	44	1819	P	44	1819	0	2.15E-17	LSC; tRNA (trnK-UUU intron)	LSC; tRNA (trnK-UUU intron)
	53	63867	P	53	63867	-3	5.18E-17	LSC; IGS (petA-psbJ)	LSC; IGS (petA-psbJ)
	49	91558	P	49	148136	-2	2.22E-16	IRb; CDS (ycf2)	IRa; CDS (ycf2)
	49	91579	P	49	148157	-2	2.22E-16	IRb; CDS (ycf2)	IRa; CDS (ycf2)
	42	37167	P	42	37167	0	3.43E-16	LSC; IGS (psbZ-trnG-UCC)	LSC; IGS (psbZ-trnG-UCC)
	36	94001	P	36	145709	0	1.41E-12	IRb; CDS (ycf2)	IRa; CDS (ycf2)
	36	94019	P	36	145727	0	1.41E-12	IRb; CDS (ycf2)	IRa; CDS (ycf2)
	36	111333	P	36	128361	0	1.41E-12	IRb; IGS (trnR-ACG- trnN-GUU)	IRa; IGS (trnN-GUU-trnR-ACG)
	36	111367	P	36	128395	0	1.41E-12	IRb; IGS (trnR-ACG- trnN-GUU)	IRa; IGS (trnN-GUU-trnR-ACG)

42	121703	P	42	121703	-2	2.66E-12	SSC; IGS (rps15-ycf1)	SSC; IGS (rps15-ycf1)
35	111649	P	35	128040	0	5.63E-12	IRb; IGS (trnR-ACG- trnN-GUU)	IRa; IGS (trnN-GUU-trnR-ACG)
35	111689	P	35	128080	0	5.63E-12	IRb; IGS (trnR-ACG- trnN-GUU)	IRa; IGS (trnN-GUU-trnR-ACG)
39	44546	P	39	138254	-2	1.47E-10	LSC; CDS (ycf3 intron2)	IRa; IGS (trnV-GAC -rps12)
32	75450	P	32	75450	0	3.60E-10	LSC; IGS (psbB-psbT)	LSC; IGS (psbB-psbT)
38	1529	P	38	1529	-2	5.56E-10	LSC; IGS (psbA-trnK-UUU)	LSC; IGS (psbA-trnK-UUU)
37	123146	P	37	123146	-3	7.38E-08	SSC; CDS (ycf1)	SSC; CDS (ycf1)
31	8640	P	31	46012	-1	1.34E-07	LSC; tRNA (trnS-GCU)	LSC; tRNA (trnS-GGA)
32	36430	P	32	46014	-3	4.82E-05	LSC; IGS, tRNA (psbC-trnS-UGA, trnS-UGA)	LSC; tRNA, IGS (trnS-GGA, trnS-GGA-rps4)
30	86302	P	30	153432	-3	6.32E-04	IRb; IGS, CDS (rpl22-rps19, rps19)	IRa; CDS, IGS (rps19, rps19-rpl22)
30	86325	P	30	153409	-3	6.32E-04	IRb; CDS (rps19)	IRa; CDS (rps19)
30	91580	P	30	148154	-3	6.32E-04	IRb; CDS (ycf2)	IRa; CDS (ycf2)
30	91601	P	30	148133	-3	6.32E-04	IRb; CDS (ycf2)	IRa; CDS (ycf2)

Table S2d The repeats distribution in *Platanthera japonica* chloroplast genome.

	Length of the 1st repeat	Starting position of the 1st repeat	Direction	Length of the 2nd repeat	Starting position of the 2nd repeat	Distance between the repeats	Evalue	Region A	Region B
Dispersed repeats	230	54479	F	230	54724	-1	1.57E-126	LSC; IGS (atpB-rncL)	LSC; IGS (atpB-rncL)
	190	63586	F	190	63780	-3	8.34E-98	LSC; IGS (petA-psbJ)	LSC; IGS (petA-psbJ)
	174	29918	F	174	30092	0	1.18E-95	LSC; IGS (petN-psbM)	LSC; IGS (petN-psbM)
	160	63616	F	160	63810	0	3.16E-87	LSC; IGS (petA-psbJ)	LSC; IGS (petA-psbJ)
	170	14366	F	170	14543	-3	6.55E-86	LSC; IGS (atpH-atpI)	LSC; IGS (atpH-atpI)
	168	118767	F	168	118995	-3	1.01E-84	SSC; IGS (pasC-ndhE)	SSC; IGS (pasC-ndhE)
	149	48298	F	149	48450	0	1.33E-80	LSC; IGS (trnL UAA intron)	LSC; IGS (trnL UAA intron)
	149	118786	F	149	119014	0	1.33E-80	SSC; IGS (psaC-ndhE)	SSC; IGS (psaC-ndhE)
	145	48916	F	145	49059	0	3.40E-78	LSC; IGS (trnL UAA-trnF GAA)	LSC; IGS (trnL UAA-trnF GAA)
	145	60853	F	145	60995	0	3.40E-78	LSC; IGS (ycf4-cemA)	LSC; IGS (ycf4-cemA)
	112	115088	F	112	115197	0	2.51E-58	LSC; IGS (ndhF-rpl32)	LSC; IGS (ndhF-rpl32)

106	126122	F	106	126227	0	1.03E-54	SSC; CDS (ycf1)	SSC; CDS (ycf1)
98	4872	F	98	4966	-1	1.98E-47	LSC; IGS (trnK UUU-rps16)	LSC; IGS (trnK UUU-rps16)
89	28441	F	89	28546	0	1.76E-44	LSC; IGS (rpoB-trnC GCA)	LSC; IGS (rpoB-trnC GCA)
87	47733	F	87	47823	0	2.82E-43	LSC; IGS (trnT UGU-trnL UAA)	LSC; IGS (trnT UGU-trnL UAA)
86	73540	F	86	73626	0	1.13E-42	LSC; IGS (clpP-psbB)	LSC; IGS (clpP-psbB)
77	0	F	77	154918	0	2.96E-37	LSC; IGS (rps19-psbA)	IRa; IGS (rps19-psbA)
77	102200	F	77	102276	0	2.96E-37	IRb; IGS (rps12-trnV GAC)	IRb; IGS (rps12-trnV GAC)
77	138621	F	77	138697	0	2.96E-37	IRa; IGS (trnV GAC-rps12)	IRa; IGS (trnV GAC-rps12)
75	105706	F	75	105777	0	4.73E-36	IRb; tRNA (trnI GAU intron)	IRb; tRNA (trnI GAU intron)
75	135122	F	75	135193	0	4.73E-36	IRa; tRNA (trnI GAU intron)	IRa; tRNA (trnI GAU intron)
73	28122	F	73	28205	0	7.57E-35	LSC; IGS (rpoB-trnC GCA)	LSC; IGS (rpoB-trnC GCA)
72	56781	F	72	56864	0	3.03E-34	LSC; IGS (rbcL-accD)	LSC; IGS (rbcL-accD)
69	59466	F	69	59555	0	1.94E-32	LSC; IGS (accD-psaI)	LSC; IGS (accD-psaI)
67	1686	F	67	1766	0	3.10E-31	IRb; tRNA (trnI GAU intron)	IRb; tRNA (trnI GAU intron)
64	57203	F	64	57279	0	1.99E-29	LSC; IGS (rbcL-accD)	LSC; IGS (rbcL-accD)
62	69784	F	62	69873	0	3.18E-28	LSC; IGS (rpl20-rps12)	LSC; IGS (rpl20-rps12)
62	86084	F	62	86144	0	3.18E-28	IRb; IGS (rpl22-rps19)	IRb; IGS (rpl22-rps19)
62	154768	F	62	154828	0	3.18E-28	IRa; IGS (rps19-psbA)	IRa; IGS (rps19-psbA)
58	49446	F	58	49520	0	8.13E-26	LSC; IGS (trnF GAA-ndhJ)	LSC; IGS (trnF GAA-ndhJ)
61	78907	F	61	78986	-1	2.33E-25	LSC; CDS (petD)	LSC; CDS (petD)
44	14818	F	44	14862	0	2.18E-17	LSC; IGS (atpH-atpI)	LSC; IGS (atpH-atpI)
47	118924	F	47	119151	-1	4.81E-17	SSC; IGS (psaC-ndhE)	SSC; IGS (psaC-ndhE)
38	123804	F	38	123852	0	8.94E-14	SSC; CDS (ycf1)	SSC; CDS (ycf1)
36	1347	F	36	1412	0	1.43E-12	LSC; IGS (psbA-trnK UUU)	LSC; IGS (psbA-trnK UUU)
36	37317	F	36	37386	0	1.43E-12	LSC; IGS (trnG UCC-trnfM CAU)	LSC; IGS (trnG UCC-trnfM CAU)
35	13974	F	35	14013	0	5.72E-12	LSC; IGS (atpF-atpH)	LSC; IGS (atpF-atpH)

	34	102951	F	34	102987	0	2.29E-11	IRb; IGS (rps12-trnV GAC)	IRb; IGS (rps12-trnV GAC)
	34	137953	F	34	137989	0	2.29E-11	IRa; IGS (trnV GAC-rps12)	IRa; IGS (trnV GAC-rps12)
	33	33614	F	33	33659	0	9.16E-11	LSC; IGS (trnT GGU-psbC)	LSC; IGS (trnT GGU-psbC)
	33	112075	F	33	112108	0	9.16E-11	IRb; IGS (trnR ACG-trnN GUU)	IRb; IGS (trnR ACG-trnN GUU)
	33	128833	F	33	128866	0	9.16E-11	IRa; IGS (trnN GUU-trnR ACG)	IRa; IGS (trnN GUU-trnR ACG)
	31	125373	R	31	125373	-2	6.13E-06	SSC; CDS (ycf1)	SSC; CDS (ycf1)
	30	42905	C	30	56710	-3	6.42E-04	LSC; IGS (psaA-ycf3)	LSC; IGS (psaA-ycf3)
Palindromic repeats	75	105706	P	75	135122	0	4.73E-36	IRb; tRNA (trnI GAU intron)	IRa; tRNA (trnI GAU intron)
	75	105777	P	75	135193	0	4.73E-36	IRb; tRNA (trnI GAU intron)	IRa; tRNA (trnI GAU intron)
	62	86084	P	62	154768	0	3.18E-28	IRb; IGS (rpl22-rps19)	IRa; IGS (rps19-psbA)
	62	86144	P	62	154828	0	3.18E-28	IRb; IGS (rpl22-rps19)	IRa; IGS (rps19-psbA)
	50	30338	P	50	30338	0	5.33E-21	LSC; IGS (petN-psbM)	LSC; IGS (petN-psbM)
	47	82	P	47	82	-1	4.81E-17	LSC; IGS (rps19-psbA)	LSC; IGS (rps19-psbA)
	34	59987	P	34	59987	0	2.29E-11	LSC; IGS (psaI-ycf4)	LSC; IGS (psaI-ycf4)
	34	102951	P	34	137953	0	2.29E-11	IRb; IGS (rps12-trnV GAC)	IRa; IGS (trnV GAC-rps12)
	34	102987	P	34	137989	0	2.29E-11	IRb; IGS (rps12-trnV GAC)	IRa; IGS (trnV GAC-rps12)
	34	120014	P	34	120014	0	2.29E-11	SSC; CDS (ndhA intron)	SSC; CDS (ndhA intron)
	33	112075	P	33	128833	0	9.16E-11	IRb; IGS (trnR ACG-trnN GUU)	IRa; IGS (trnN GUU-trnR ACG)
	33	112108	P	33	128866	0	9.16E-11	IRb; IGS (trnR ACG-trnN GUU)	IRa; IGS (trnN GUU-trnR ACG)
	39	122393	P	39	122393	-3	5.52E-09	SSC; IGS (rps15-ycf1)	SSC; IGS (rps15-ycf1)
	33	8673	P	33	45965	-1	9.07E-09	LSC; tRNA (trnS GCU)	LSC; tRNA (trnS GGA)
	31	108001	P	31	132559	-1	1.36E-07	IRb; rRNA (rrn 23)	IRa; rRNA (rrn 23)
	31	108384	P	31	132942	-1	1.36E-07	IRb; rRNA (rrn 23)	IRa; rRNA (rrn 23)
	34	107965	P	34	132592	-3	3.70E-06	IRb; rRNA (rrn 23)	IRa; rRNA (rrn 23)
	34	107985	P	34	132572	-3	3.70E-06	IRb; rRNA (rrn 23)	IRa; rRNA (rrn 23)
	34	108348	P	34	132975	-3	3.70E-06	IRb; rRNA (rrn 23)	IRa; rRNA (rrn 23)

	34	108368	P	34	132955	-3	3.70E-06	IRb; rRNA (rrn 23)	IRa; rRNA (rrn 23)
	33	36427	P	33	45964	-3	1.35E-05	LSC; tRNA (trnS UGA)	LSC; tRNA (trnS GGA)

Table S3 The tandem repeats distribution in the chloroplast genomes of four Orchidaceae species.

Table S3a The tandem repeats distribution in *Cremastra appendiculata* chloroplast genome.

Tandem repeats	Indices	Size(bp)	Repeat number	Start position	Region
	3825--4006	90	2	3825	LSC;tRNA(trnK-UUU intron)
	6139--6265	61	2	6139	LSC;IGS(rps16-trnQ-UUG)
	7061--7175	50	2	7061	LSC;IGS(rps16-trnQ-UUG)
	7838--8053	107	2	7838	LSC;IGS(trnQ-UUG-psbK)
	8605--8717	53	2	8605	LSC;IGS(psbK-psbI)
	8922--9075	67	2	8922	LSC;IGS(psbI-trnS-GCU)
	9496--9592	38	2	9496	LSC;IGS(trnS-GCU-trnG-UCC)
	9858--9957	43	2	9858	LSC;IGS(trnS-GCU-trnG-UCC)
	13876--13971	47	2	13876	LSC;CDS(atpF intron)
	23668--23780	54	2	23668	LSC;CDS(rpoC1 intron)
	29385--29678	146	2	29385	LSC;IGS(trnC-GCA-petN)
	30109--30514	203	2	30109	LSC;IGS(petN-psbM)
	33435--33576	70	2	33435	LSC;IGS(trnT-GGU-psbD)
	34078--34245	78	2	34078	LSC;IGS(trnT-GGU-psbD)
	37460--37647	95	2	37460	LSC;IGS(lhbA-trnG-GCC)
	37831--37958	62	2	37831	LSC;IGS(trnG-GCC-trnfM-CAU)
	43517--43610	39	2	43517	LSC;IGS(psaA-ycf3)
	44684--44878	97	2	44684	LSC;CDS(ycf3 intron)
	46733--46870	69	2	46733	LSC;IGS(trnS-GGA-rps4)
	48079--48337	127	2	48079	LSC;IGS(trnT-UGU-trnL-UAA)
	49135--49185	23	2	49135	LSC;tRNA(trnL-UAA intron)
	50245--50443	100	2	50245	LSC;IGS(trnF-GAA-ndhJ)
	50519--50820	152	2	50519	LSC;IGS(trnF-GAA-ndhJ)
	55437--55553	52	2	55437	LSC;IGS(atpB-rbcL)
	64569--64789	109	2	64569	LSC;IGS(petA-psbJ)
	67079--67237	51	3	67079	LSC;IGS(psbE-petL)
	67039--67238	99	2	67039	LSC;IGS(psbE-petL)
	70778--70885	54	2	70778	LSC;IGS(rpl20-clpP)
	71211--71390	85	2	71211	LSC;IGS(rpl20-clpP)
	79705--79776	35	2	79705	LSC;IGS(petB-petD)
	89311--89357	22	2	89311	IRB;IGS(rpl2-rpl23)
	89517--89567	25	2	89517	IRB;IGS,CDS(rpl2-rpl23,rpl23)
	96953--97003	25	2	96953	IRB;CDS(ycf2)
	103493--103689	98	2	103493	IRB;IGS(rps7-trnV-GAC)
	115738--115922	93	2	115738	SSC;IGS(rpl32-trnL-UAG)
	116242--116398	65	2	116242	SSC;IGS(rpl32-trnL-UAG)
	122549--122749	99	2	122549	SSC;IGS(ndhH-rps15)
	123328--123447	58	2	123328	SSC;IGS(rps15-ycf1)
	124252--124416	50	3	124252	SSC;IGS(rps15-ycf1)
	124264--124464	98	2	124264	SSC;IGS(rps15-ycf1)
	125503--125727	112	2	125503	SSC;IGS,CDS(rps15-ycf1,ycf1)
	138730--138926	98	2	138730	IRA;IGS(trnV-GAC-rps7)
	145416--145466	25	2	145416	IRA;CDS(ycf2)
	152852--152902	25	2	152852	IRA;IGS,CDS(rpl23-rpl2,rpl23)

	153062--153108	22	2	153062	IRA;IGS (rpl23-rpl2)

Table S3b The tandem repeats distribution in *Calanthe davidii* chloroplast genome.

Tandem repeats	Indices	Size (bp)	Repeat number	Start position	Region
	14048--14168	59	2	14048	LSC; IGS (atpF-atpH)
	14987--15031	22	2	14987	LSC; IGS (atpH-atpI)
	32463--32771	156	2	32463	LSC; IGS (trnE-UUC-trnT-GGU)
	33650--33813	78	2	33650	LSC; IGS (trnT-GGU-psbD)
	42718--42908	96	2	42718	LSC; IGS (psaA-ycf3)
	45483--45705	111	2	45483	LSC; IGS (ycf3-trnS-GGA)
	47551--47718	79	2	47551	LSC; IGS (trnT-UGU-trnL-UAA)
	48284--48750	233	2	48284	LSC; tRNA (trnL-UAA intron)
	49995--50116	45	3	49995	LSC; IGS (trnF-GAA-ndhJ)
	50951--51138	94	2	50951	LSC; CDS (ndhK)
	54836--55090	116	2	54836	LSC; IGS (atpB-rbcL)
	62488--62678	95	2	62488	LSC; CDS, IGS (cemA, cemA-petA)
	73673--73787	50	2	73673	LSC; IGS (clpP-psbB)
	83771--83920	75	2	83771	LSC; CDS (rpl16 intron)
	86181--86266	39	2	86181	IRb; IGS (rpl22-rps19)
	91116--91219	52	2	91116	IRb; IGS, CDS (trnI-CAU-ycf2, ycf2)
	93946--94006	18	4	93946	IRb; CDS (ycf2)
	102319--102372	21	3	102319	IRb; IGS (rps12-trnV-GAC)
	102303--102380	35	2	102303	IRb; IGS (rps12-trnV-GAC)
	111167--111312	72	2	111167	IRb; IGS (trnR-ACG-trnN-GUU)
	114519--114708	93	2	114519	SSC; IGS (rpl32-trnL-UAG)
	114960--115085	58	2	114960	SSC; IGS (rpl32-trnL-UAG)
	123665--123790	63	2	123665	SSC; CDS (ycf1)
	128363--128508	72	2	128363	IRa; IGS (trnN-GUU-trnR-ACG)
	137303--137356	21	3	137303	IRa; IGS (trnV-GAC-rps12)
	137295--137372	35	2	137295	IRa; IGS (trnV-GAC-rps12)
	145669--145729	18	4	145669	IRa; CDS (ycf2)
	148456--148559	52	2	148456	IRb; CDS, IGS (ycf2, ycf2-trnI-CAU)
	153409--153494	39	2	153409	IRa; IGS (rps19-rpl22)

Table S3c The tandem repeats distribution in *Epipactis mairei* chloroplast genome.

Tandem repeats	Indices	Size(bp)	Repeat number	Start position	Region
	3431--3812	190	2	3431	LSC; tRNA (trnK-UUU intron)
	6689--6835	71	2	6689	LSC; IGS (rps16-trnQ-UUG)
	7906--8135	110	2	7906	LSC; IGS (psbK-psbI)
	8952--9567	307	2	8952	LSC; IGS (trnS-GCU-trnG-GCC)
	13135--13449	155	2	13135	LSC; CDS (atpF intron)
	13980--14099	60	2	13980	LSC; IGS (atpF-atpH)
	14356--14512	82	2	14356	LSC; CDS, IGS (atpH, atpH-atpI)
	14939--15022	41	2	14939	LSC; IGS (atpH-atpI)
	16079--16243	80	2	16079	LSC; IGS, CDS (atpI-rps2, rps2)

	28202--28501	151	2	28202	LSC; IGS (rpoB-trnC-GCA)
	29829--29974	63	2	29829	LSC; IGS (petN-psbM)
	32314--32571	128	2	32314	LSC; IGS (trnE-UUC-trnT-GGU)
	33547--33681	68	2	33547	LSC; IGS (trnT-GGU-psbD)
	43565--43742	88	2	43565	LSC; CDS (ycf3 intron1)
	45801--45908	41	3	45801	LSC; IGS (ycf3-trnS-GGA)
	48352--48666	157	2	48352	LSC; tRNA (trnL-UAA intron)
	54702--54835	67	2	54702	LSC; IGS (atpB-rbcL)
	56812--56960	74	2	56812	LSC; IGS (rbcL-accD)
	59081--59440	181	2	59081	LSC; IGS (accD-psaI)
	61525--61615	47	2	61525	LSC; IGS (ycf4-cemA)
	64158--64309	77	2	64158	LSC; IGS (petA-psbJ)
	64354--64446	45	2	64354	LSC; IGS (petA-psbJ)
	65756--65953	90	2	65756	LSC; IGS (psbE-petL)
	68125--68346	104	2	68125	LSC; IGS (psaJ-rpl33)
	73317--73375	26	2	73317	LSC; IGS (clpP-psbB)
	75451--75754	136	2	75451	LSC; IGS (psbB-psbT)
	78943--79042	49	2	78942	LSC; CDS (petD intron)
	83837--83932	45	2	83837	LSC; CDS (rpl16 intron)
	86308--86353	23	2	86377	IRb; CDS (rps19)
	91539--91628	21	4	91539	IRb; CDS (ycf2)
	91560--91628	21	3	91560	IRb; CDS (ycf2)
	93990--94055	18	4	93990	IRb; CDS (ycf2)
	95902--95995	47	2	95902	IRb; IGS (ycf2-ycf15)
	99898--100099	100	2	99898	IRb; IGS (ndhB-rps7)
	102398--102513	54	2	102398	IRb; IGS (rps12-trnV-GAC)
	102400--102534	54	3	102400	IRb; IGS (rps12-trnV-GAC)
	111334--111403	34	2	111334	IRb; IGS (trnR-ACG-trnN-GUU)
	114531--114643	47	2	114531	SSC; IGS (rpl32-trnL-UAG)
	114539--114655	47	3	114539	SSC; IGS (rpl32-trnL-UAG)
	115152--115285	62	2	115152	SSC; IGS (trnL-UAG-ccsA)
	121174--121290	52	2	121174	SSC; IGS (ndhH-rps15)
	122640--122882	122	2	122640	SSC; CDS (ycf1)
	125537--125699	81	2	125537	SSC; CDS (ycf1)
	128362--128431	34	2	128362	IRa; IGS (trnN-GUU-trnR-ACG)
	137252--137367	54	2	137252	IRa; IGS (trnV-GAC-rps12)
	139666--139867	100	2	139666	IRa; IGS (rps7-ndhB)
	143770--143863	47	2	143770	IRa; IGS (ycf15-ycf2)
	145710--145775	18	4	145710	IRa; CDS (ycf2)
	148137--148205	21	3	148137	IRa; CDS (ycf2)
	148137--148226	21	4	148137	IRa; CDS (ycf2)
	153412--153457	23	2	153412	IRa; CDS (rps19)

Table S3d The tandem repeats distribution in *Platanthera japonica* chloroplast genome.

Tandem repeats	Indices	Size(bp)	Repeat number	Start position	Region
	1339--1466	65	2	1339	LSC; IGS (psbA-trnK UUU)
	1687--1845	80	2	1687	LSC; tRNA (trnK UUU)

4873--5064	94	2	4873	LSC; IGS (trnK UUU-rps16)
7984--8084	50	2	7984	LSC; IGS (psbK-psbI)
8942--9781	420	2	8942	LSC; IGS (trnS GCU-trnG GCC)
13168--13242	37	2	13168	LSC; CDS (atpF intron)
13965--14053	39	2	13965	LSC; IGS (atpF-atpH)
14367--14720	177	2	14367	LSC; IGS (atpH-atpI)
14819--14906	44	2	14819	LSC; IGS (atpH-atpI)
16015--16088	37	2	16015	LSC; IGS (atpI-rps2)
28123--28287	83	2	28123	LSC; IGS (rpoB-trnC GCA)
28433--28642	105	2	28433	LSC; IGS (rpoB-trnC GCA)
29811--29878	32	2	29811	LSC; CDS, IGS (petN, petN-psbM)
29919--30266	174	2	29919	LSC; IGS (petN-psbM)
31040--31539	250	2	31040	LSC; IGS (psbM-trnD GUC)
33615--33700	45	2	33615	LSC; IGS (trnD GUC-psbD)
37304--37438	69	2	37304	LSC; IGS (trnG UCC-trnfM CAU)
46934--47026	44	2	46934	LSC; IGS (rps4-trnT UGU)
47729--47921	90	2	47729	LSC; IGS (trnT UGU-trnL UAA)
48299--48599	152	2	48299	LSC; tRNA (trnL UAA intron)
48917--49204	143	2	48917	LSC; IGS (trnL UAA-trnF GAA)
49447--49595	74	2	49447	LSC; IGS (trnF GAA-ndhJ)
54480--54954	245	2	54480	LSC; IGS (atpB-rbcL)
56782--56950	83	2	56782	LSC; IGS (rbcL-accD)
57204--57357	76	2	57204	LSC; IGS (rbcL-accD)
59467--59643	89	2	59467	LSC; IGS (accD-psaI)
60854--61140	142	2	60854	LSC; IGS (ycf4-cemA)
63587--63970	194	2	63587	LSC; IGS (petA-psbJ)
69771--69953	89	2	69771	LSC; IGS (rpl20-rps12)
72570--72683	55	2	72570	LSC; CDS (clpP intron2)
73534--73712	86	2	73534	LSC; IGS (clpP-psbB)
78893--79060	79	2	78893	LSC; CDS (petD intron)
86085--86206	60	2	86085	IRb; IGS (rpl22-rps19)
93362--93444	42	2	93362	IRb; CDS (ycf2)
93908--93967	24	2	93908	IRb; CDS (ycf2)
101395--102148	378	2	101395	IRb; IGS (rps12-trnV GAC)
102201--102353	76	2	102201	IRb; IGS (rps12-trnV GAC)
102952--103021	36	2	102952	IRb; IGS (rps12-trnV GAC)
105707--105852	71	2	105707	IRb; tRNA (trnI GAU intron)
112076--112141	33	2	112076	IRb; IGS (trnR ACG-trnN GUU)
114400--114485	36	2	114400	SSC; IGS (ndhF-rpl32)
115089--115309	109	2	115089	SSC; IGS (ndhF-rpl32)
115855--115951	46	2	115855	SSC; IGS (trnL UAG-ccsA)
123805--123899	48	2	123805	SSC; CDS (ycf1)
126123--126338	105	2	126123	SSC; CDS (ycf1)
127248--127393	69	2	127248	SSC; CDS (ycf1)
128834--128899	33	2	128834	IRa; IGS (trnN GUU-trnR ACG)
135123--135268	71	2	135123	IRa; tRNA (trnI GAU intron)
137954--138023	36	2	137954	IRa; IGS (trnV GAC-rps12)
138622--138774	76	2	138622	IRa; IGS (trnV GAC-rps12)
138827--139580	378	2	138827	IRa; IGS (trnV GAC-rps12)

147008--147067	24	3	147008	IRa; CDS (ycf2)
154769--154890	60	2	154769	IRa CDS (ycf2)

Table S4 Simple sequence repeats in the chloroplast genomes of four Orchidaceae species.

Table S4a Simple sequence repeats in *Cremastra appendiculata* chloroplast genome.

SSR type	SSR	size	start	end	Location	Region
p1	(T)10	10	2140	2149	trnK-UUU intron	LSC
p1	(A)11	11	6854	6864	rps16-trnQ-UUG IGS	LSC
p1	(T)11	11	10358	10368	trnG-GCC intron	LSC
p1	(T)12	12	29783	29794	trnC-GCA-petN IGS	LSC
p1	(A)11	11	33336	33346	trnT-GGU-psbD IGS	LSC
p1	(A)10	10	45790	45799	ycf3 intron2	LSC
p1	(T)10	10	55767	55776	atpB-rbcL IGS	LSC
p1	(A)10	10	57646	57655	rbcL-accD IGS	LSC
p1	(A)10	10	65870	65879	psbF CDS	LSC
p1	(T)10	10	70290	70299	rps18-rpl20, rpl20 IGS-CDS	LSC
p1	(T)11	11	72335	72345	clpP intron2	LSC
p1	(T)10	10	104181	104190	rps12-trnV-GAC IGS	IRb
p1	(T)10	10	115837	115846	rpl32-trnL-UAG IGS	SSC
p1	(A)10	10	126330	126339	ycf1 CDS	SSC
p1	(T)10	10	127729	127738	ycf1 CDS	SSC
p1	(A)12	12	127890	127901	ycf1 CDS	SSC
p1	(A)10	10	138229	138238	trnV-GAC-rps12 IGS	IRa
p2	(AT)5	10	522	531	rps19-psbA IGS	LSC
p2	(TA)5	10	11191	11200	trnR-UCU-atpA	LSC
p2	(AT)5	10	20954	20963	rpoC2 CDS	LSC
p2	(TA)5	10	30178	30187	petN-psbM IGS	LSC
p2	(TA)5	10	30381	30390	petN-psbM IGS	LSC
p2	(AT)5	10	30579	30588	petN-psbM IGS	LSC
p2	(AT)5	10	43553	43562	psaA-ycf3 IGS	LSC
p2	(TA)5	10	44368	44377	ycf3 intron1	LSC
p2	(AT)9	18	48117	48134	trnT-UGU-trnL-UAA IGS	LSC
p2	(AT)9	18	48244	48261	trnT-UGU-trnL-UAA IGS	LSC
p2	(AT)5	10	48391	48400	trnT-UGU-trnL-UAA IGS	LSC
p2	(AT)6	12	61877	61888	ycf4-cemA IGS	LSC
p2	(TA)6	12	74099	74110	clpP-psbB IGS	LSC
p2	(TA)6	12	76323	76334	psbB-psbT IGS	LSC
p2	(TA)7	14	83427	83440	rps8-rpl14 IGS	LSC
p2	(GA)5	10	93338	93347	ycf2 CDS	IRb
p2	(TA)8	16	119556	119571	psaC-ndhE IGS	SSC
p2	(TC)5	10	149072	149081	ycf2 CDS	IRa
p4	(ACTA)3	12	7007	7018	rps16-trnQ-UUG IGS	LSC
p4	(GTCT)3	12	12350	12361	atpA CDS	LSC
p4	(ATTA)3	12	69028	69039	psaJ-rpl33 IGS	LSC
p4	(AAAT)3	12	70009	70020	rps18,rps18-rpl20 CDS-IGS	LSC
p5	(TTACT)3	15	37536	37550	psbZ-trnG-UCC IGS	LSC
p5	(TTCTA)3	15	73478	73492	clpP intron2	LSC
p6	(TATTTA)3	18	36801	36818	psbC-trnS-UGA IGS	LSC
c	(A)11ggaagttttttttcatctta<br>tatcattatagaatgaaatttc<br>(A)11ggaagttttttttcatctt	174	8631	8804	psbK-psbI IGS	LSC

c	tatctttagagtgtcatatcaaa Atagtgtatagtgtgtgtcgtaa aataggtgatctatttccttaaa aaaaatg(ATCTT)3 (TA)10tcagtcaagtgaatc ttgttctgatggaagagtcaa caaggttaatcttgaaacaattc aatgcatttc(T)10	99	32976	33074	trnE-UUC-trnT-GGU IGS	LSC
c	(TA)6tctttctatctttaatat ttttatctttactattagatat(TA)6	64	55452	55515	atpB-rbcL IGS	LSC
c	(AAT)4acaatataaatataga agaaatag(AATA)3	48	76451	76498	psbB-psbT IGS	LSC
c	(A)12taaaaaagaacaagga taataagaaactcaaagaaga aatgaaattttaattaacgagtttc atgaatttcttaaaatgaaaaatct(A)12	111	122578	122688	ndhH-rps15 IGS	SSC

Table S4b Simple sequence repeats in *Calanthe davidii* chloroplast genome.

SSR type	SSR	size	start	end	Location	Region
p1	(T)10	10	1568	1577	psbA-trnK-UUU IGS	LSC
p1	(T)10	10	1858	1867	trnK-UUU intron	LSC
p1	(A)11	11	6553	6563	rps16-trnQ-UUG IGS	LSC
p1	(A)10	10	9586	9595	trnS-GCU-trnG-GCC IGS	LSC
p1	(A)10	10	16009	16018	atpI-rps2 IGS	LSC
p1	(T)12	12	16910	16921	rps2-rpoC2 IGS	LSC
p1	(T)10	10	29288	29297	trnC-GCA-petN IGS	LSC
p1	(A)10	10	32996	33005	trnT-GGU-psbD IGS	LSC
p1	(T)11	11	43147	43157	psaA-ycf3 IGS	LSC
p1	(T)10	10	49010	49019	trnL-UAA-trnF-GAA IGS	LSC
p1	(A)10	10	51167	51176	ndhC CDS	LSC
p1	(A)10	10	59223	59232	accD CDS	LSC
p1	(A)13	13	61198	61210	ycf4-cemA IGS	LSC
p1	(T)10	10	63846	63855	petA-psbJ IGS	LSC
p1	(T)10	10	66536	66545	psbE-petL IGS	LSC
p1	(A)11	11	77142	77152	petB intron	LSC
p1	(T)11	11	78533	78543	petD intron	LSC
p1	(T)17	17	81626	81642	rpl36-infA IGS	LSC
p1	(T)10	10	82534	82543	rps8-rpl14 IGS	LSC
p1	(T)12	12	84789	84800	rpl16 intron	LSC
p1	(T)10	10	116462	116471	ccsA-ndhD IGS	SSC
p1	(A)11	11	119047	119057	ndhG-ndhI	SSC
p1	(T)10	10	122821	122830	ycf1 CDS	SSC
p1	(A)10	10	124816	124825	ycf1 CDS	SSC
p1	(T)14	14	126130	126143	ycf1 CDS	SSC
p1	(T)10	10	126251	126260	ycf1 CDS	SSC
p2	(TA)7	14	5564	5577	rps16 intron	LSC
p2	(AT)5	10	20506	20515	rpoC2 CDS	LSC
p2	(TA)5	10	54864	54873	atpB-rbcL IGS	LSC
p2	(TA)5	10	54980	54989	atpB-rbcL IGS	LSC

p2	(TA)5	10	64339	64348	petA-psbJ IGS	LSC
p2	(TA)6	12	75757	75768	psbB-psbT IGS	LSC
p2	(GA)5	10	92159	92168	ycf2 CDS	IRb
p2	(TC)5	10	147507	147516	ycf2 CDS	IRa
p3	(TTC)4	12	3634	3645	trnK-UUU intron	LSC
p3	(AAT)4	12	118442	118453	psaC-ndhE IGS	SSC
p4	(GTCT)3	12	11884	11895	atpA CDS	LSC
p4	(TTTA)3	12	28577	28588	rpoB-trnC-GCA IGS	LSC
p4	(ATTA)3	12	68307	68318	psaJ-rpl33 IGS	LSC
p4	(TTTA)3	12	79739	79750	petD-rpoA IGS	LSC
p4	(TTGA)3	12	118604	118615	ndhE CDS	SSC
p5	(ATCTT)3	15	8330	8344	trnS-GCU-trnG-GCC IGS	LSC
c	(TCTA)3ttatctat(ATAG)4	36	5356	5391	rps16 intron	LSC
c	(T)10ccattcctggtggtatcaaaa Tgccactgtgcctagatattttatccac Ctctccagaaaaatgaatatctccaga aaatattttcagttccatac(T)11	117	19021	19137	rpoC2 CDS	LSC
c	(AT)5cataatggaagatgaagatgc ctaattcatgaattg(A)11	57	32799	32855	trnE-UUC-trnT-GGU IGS	LSC
c	(CTA)4aaatttctat(ACAAA)3	37	51331	51367	ndhC-trnV-UAC IGS	LSC
c	(AATG)3aataatttattagatcttttt Ctttatacttctaccttatttacttcaaggt Attcttagctatagtagtaattctatcattc (AATG)3	107	62505	62611	cemA, cemA-petA CDS-IGS	LSC
c	(TG)5atttttccaagtagcgggaaga Gtaagaactttacatcttaacatatataat Acaaaccaaatcctattttggtcgaatct taaatgaataag(A)10	113	68800	68912	rpl33-rps18 IGS	LSC
c	(A)10tcaatgtattgattccagtcctatt tc(T)11	48	72213	72260	clpP intron2	LSC
c	(T)11ccatcactcgtacatgg(A)12	40	126384	126423	ycf1 CDS	SSC

Table S4c Simple sequence repeats in *Epipactis mairei* chloroplast genome.

SSR type	SSR	size	start	end	Location	Region
p1	(A)13	13	4055	4067	trnK-UUU intron	LSC
p1	(A)10	10	4946	4955	trnK-UUU-rps16 IGS	LSC
p1	(A)11	11	8247	8257	psbK-psbI IGS	LSC
p1	(T)11	11	9881	9891	trnG-GCC intron	LSC
p1	(A)13	13	14778	14790	atpH-atpI IGS	LSC
p1	(T)11	11	23662	23672	rpoC1 intron	LSC
p1	(A)12	12	32741	32752	trnE-UUC-trnT-GGU IGS	LSC
p1	(A)12	12	32894	32905	trnT-GGU-psbD IGS	LSC
p1	(T)11	11	36325	36335	psbC-trnS-UGA IGS	LSC
p1	(T)11	11	36682	36692	trnS-UGA-psbZ IGS	LSC
p1	(A)11	11	47634	47644	trnT-UGU-trnL-UAA IGS	LSC
p1	(A)11	11	48199	48209	trnL-UAA intron	LSC
p1	(T)10	10	48965	48974	trnL-UAA-trnF-GAA IGS	LSC
p1	(T)10	10	56772	56781	rbcL-accD IGS	LSC
p1	(T)10	10	57318	57327	rbcL-accD IGS	LSC

p1	(T)11	11	60176	60186	psaI-ycf4 IGS	LSC
p1	(T)12	12	67519	67530	trnP-UGG-psaJ IGS	LSC
p1	(A)12	12	68333	68344	psaJ-rpl33 IGS	LSC
p1	(A)11	11	71643	71653	clpP intron1	LSC
p1	(T)14	14	72450	72463	clpP intron2	LSC
p1	(T)12	12	81639	81650	rpl36-infA IGS	LSC
p1	(T)10	10	84593	84602	rpl16 intron	LSC
p1	(T)10	10	84789	84798	rpl16 intron	LSC
p1	(A)10	10	84940	84949	rpl16-rps3 IGS	LSC
p1	(T)12	12	85709	85720	rps3-rpl22 IGS	LSC
p1	(T)14	14	103084	103097	rps12-trnV-GAC IGS	IRb
p1	(T)13	13	114011	114023	ndhF-rpl32 IGS	SSC
p1	(T)10	10	115830	115839	ccsA CDS	SSC
p1	(T)11	11	119339	119349	ndhA intron	SSC
p1	(T)10	10	126209	126218	ycf1 CDS	SSC
p1	(A)12	12	126370	126381	ycf1 CDS	SSC
p1	(A)14	14	136668	136681	trnV-GAC-rps12 IGS	IRa
p2	(AT)5	10	20557	20566	rpoC2 CDS	LSC
p2	(TA)5	10	47093	47102	rps4-trnT-UGU IGS	LSC
p2	(AT)5	10	73469	73478	clpP-psbB IGS	LSC
p2	(TA)7	14	75698	75711	psbB-psbT IGS	LSC
p2	(GA)5	10	92203	92212	ycf2 CDS	IRb
p2	(TC)5	10	147553	147562	ycf2 CDS	IRa
p3	(ATT)4	12	84343	84354	rpl16 intron	LSC
p4	(AAAT)3	12	9107	9118	trnS-GCU-trnG-GCC IGS	LSC
p4	(AAAT)3	12	9414	9425	trnS-GCU-trnG-GCC IGS	LSC
p4	(TCTA)3	12	10700	10711	trnR-UCU-atpA IGS	LSC
p4	(GTCT)3	12	11865	11876	atpA CDS	LSC
p4	(AATG)3	12	62383	62394	cemA CDS	LSC
p4	(TAAA)3	12	73693	73704	clpP-psbB IGS	LSC
p4	(GAAT)3	12	123626	123637	ycf1 CDS	SSC
p5	(TTCTA)3	15	114623	114637	rpl32-trnL-UAG IGS	SSC
c	(A)11gaaatc(T)11	28	1535	1562	psbA-trnK-UUU IGS	LSC
c	(T)10catg(A)10	24	1830	1853	trnK-UUU intron	LSC
c	(TATC)3gatagataaatgtttatcc Ggaaagtatttcacttaaattaaccccat atttttcatatgaagaaaataacatg(A) 10caaatcagttttaagcaaacttatttac Atcttcaacagatctttcgggattgtcca tcataaaaatccctct(A)11	181	6331	6511	rps16-trnQ-UUG IGS	LSC
c	(A)10gtaaattcaataatgacttacaac aacc(A)15gacttataactttagtacac tatttcaatttgactaaactttttgta(T)10	110	9573	9682	trnS-GCU-trnG-GCC IGS	LSC
c	(TCT)4taatgataagatattcaaaaca Tagttcatataactccaacaaagagagttc accaaaaacgaactaa(TCT)4	92	16134	16225	atpI-rps2 IGS	LSC
c	(T)10ccattcctggtggtataacaatgc Cactgtgcctagatattttatccacctctcc Aggaaaatgaatatctccagaaaaaatttt	117	19063	19179	rpoC2 CDS	LSC

c	cagttccatac(T)11 (T)10a(CT)5	21	71482	71502	clpP intron1	LSC
c	(TA)16ttttttatgtataatataaatagatat aaatcaaaagaaatatagaagaaatagaat agtaataagatatgactataac(TA)7	125	75451	75575	psbB-psbT IGS	LSC
c	(T)21aattatgaaattataaaaaattattag aaattaaaagtatatgcatgagacacaatct attaatcggatctctttcagatatttgaatattc tattattc(TA)5	131	82537	82667	rps8-rpl14 IGS	LSC
c	(CAT)4gtattagaatgttacacagtgtata tatttttactttcatccaccaaatatgttacaag atatgtaggaaatacactataa(T)13	106	121830	121935	rps15-ycf1 IGS	SSC
c	(TTCT)3ttaaaaattttataacttgtaaaa attgctttttaacctttttta(T)11gagttcttta gaaataggttc(A)10	98	124653	124750	ycf1 CDS	SSC

Table S4d Simple sequence repeats in *Platanthera japonica* chloroplast genome.

SSR type	SSR	size	start	end	Location	Region
p1	(T)12	12	1478	1489	psbA-trnK UUU IGS	LSC
p1	(A)12	12	1646	1657	trnK UUU intron	LSC
p1	(T)11	11	4429	4439	trnK UUU intron	LSC
p1	(T)11	11	6423	6433	rps16-trnQ UUG IGS	LSC
p1	(T)11	11	7058	7068	rps16-trnQ UUG IGS	LSC
p1	(A)16	16	7581	7596	trnQ UUG-psbK IGS	LSC
p1	(T)11	11	9277	9287	trnS GCU-trnG GCC IGS	LSC
p1	(T)11	11	9697	9707	trnS GCU-trnG GCC IGS	LSC
p1	(T)11	11	9906	9916	trnG GCC intron	LSC
p1	(A)13	13	13156	13168	atpF intron	LSC
p1	(A)12	12	29781	29792	petN CDS	LSC
p1	(A)10	10	30969	30978	psbM-trnD GUC IGS	LSC
p1	(A)14	14	32463	32476	trnE UUC-trnT GGU IGS	LSC
p1	(A)10	10	32926	32935	trnT GGU-psbD IGS	LSC
p1	(A)11	11	37461	37471	trnG UCC-trnfM CAU IGS	LSC
p1	(A)16	16	38138	38153	rps14-psaB IGS	LSC
p1	(T)10	10	42910	42919	psaA CDS	LSC
p1	(T)10	10	43064	43073	psaA-ycf3 IGS	LSC
p1	(T)11	11	45103	45113	ycf3 intron	LSC
p1	(A)10	10	46928	46937	rps4-trnT UGU IGS	LSC
p1	(A)10	10	47510	47519	trnT UGU-trnL UAA IGS	LSC
p1	(A)13	13	48139	48151	trnL UAA intron	LSC
p1	(A)11	11	51728	51738	trnV UAC intron	LSC
p1	(A)10	10	56715	56724	rbcL-accD IGS	LSC
p1	(A)10	10	57444	57453	rbcL-accD IGS	LSC
p1	(A)12	12	58986	58997	accD-psaI IGS	LSC
p1	(T)10	10	67229	67238	trnP UGG-psaJ IGS	LSC
p1	(T)11	11	67348	67358	trnP UGG-psaJ IGS	LSC
p1	(A)15	15	68263	68277	psaJ-rpl33 IGS	LSC
p1	(A)10	10	68622	68631	rpl33-rps18 IGS	LSC
p1	(T)10	10	69187	69196	rps18-rpl20 IGS	LSC

p1	(T)10	10	70496	70505	rpl20-rps12 IGS	LSC
p1	(A)11	11	71218	71228	clpP intron1	LSC
p1	(T)12	12	72368	72379	clpP intron2	LSC
p1	(T)14	14	76108	76121	psbN-psbH IGS	LSC
p1	(A)10	10	77056	77065	psbH-petB IGS	LSC
p1	(A)10	10	79747	79756	petD-rpoA IGS	LSC
p1	(T)16	16	81597	81612	rpl36-infA IGS	LSC
p1	(A)11	11	84032	84042	rpl16 intron	LSC
p1	(T)11	11	84693	84703	rpl16 intron	LSC
p1	(T)10	10	85433	85442	rps3 CDS	LSC
p1	(T)11	11	116518	116528	ccsA CDS	SSC
p1	(T)11	11	117140	117150	ccsA-ndhD IGS	SSC
p1	(T)13	13	118927	118939	psaC-ndhE IGS	SSC
p1	(T)12	12	119155	119166	psaC-ndhE IGS	SSC
p1	(T)12	12	123590	123601	ycf1 CDS	SSC
p1	(T)10	10	124414	124423	ycf1 CDS	SSC
p1	(A)10	10	124739	124748	ycf1 CDS	SSC
p1	(T)10	10	125218	125227	ycf1 CDS	SSC
p1	(T)15	15	126773	126787	ycf1 CDS	SSC
p1	(T)10	10	126894	126903	ycf1 CDS	SSC
p1	(A)12	12	127049	127060	ycf1 CDS	SSC
p2	(TA)5	10	9069	9078	trnS GCU-trnG GCC IGS	LSC
p2	(TA)5	10	9489	9498	trnS GCU-trnG GCC IGS	LSC
p2	(AT)5	10	20477	20486	rpoC2 CDS	LSC
p2	(TA)6	12	30358	30369	petN-psbM IGS	LSC
p2	(TA)6	12	32303	32314	trnE UUC-trnT GGU IGS	LSC
p2	(AT)5	10	32726	32735	trnE UUC-trnT GGU IGS	LSC
p2	(AT)6	12	60002	60013	psaI-ycf4 IGS	LSC
p2	(TA)8	16	72680	72695	clpP intron2	LSC
p2	(TA)8	16	83730	83745	rpl16 intron	LSC
p2	(GA)5	10	92127	92136	ycf2 CDS	IRb
p2	(TC)5	10	148845	148854	ycf2 CDS	IRa
p3	(TAT)4	12	64266	64277	petA-psbJ IGS	LSC
p3	(ATA)4	12	115846	115857	trnL UAG-ccsA IGS	SSC
p4	(TCTA)3	12	10717	10728	trnR UCU-atpA IGS	LSC
p4	(GTCT)3	12	11853	11864	atpA CDS	LSC
p4	(AATG)3	12	62173	62184	cemA, cemA-petA CDS-IGS	LSC
c	(T)18attctttg(ATA)4	38	3627	3664	trnE UUC intron	LSC
c	(TA)5tggcaagaatttttggtaacgaaaaaaatacgaatctttttatatgattatgataatgatatttatacgagaaaggaattcatgaaaacaaat(A)14ttattatttttagagcgtcatatcaaaataatgtatagtgtgtgtcgtaaaataggtgatttatttcctt(A)10	197	8196	8392	psbK-psbI IGS	LSC
c	(T)10gtaggtgcgatccgttggacatagatccaatttttaaatttttttgatcctttagaa(T)10ccattcctggtggtatcaaaataccgccgtgcctagatatttta	184	18916	19099	rpoC2 CDS	LSC

	tccatctccccaggaaaatgaatatctc cagaaaaaattttcagttccatac(T)11					
c	(T)10atctttttatattctattatattctat atcatattc(ATT)5	61	54627	54687	atpB-rbcL IGS	LSC
c	(T)10atctttttatattctattatattctat atcatattc(ATT)5	61	54872	54932	atpB-rbcL IGS	LSC
c	(T)10atagtctttctatttatgaaaattta tattgatttatattgaattcgtgagatgattt tttctttactggattcaaagtactaagtatt gtgtatttta(T)10	119	115129	115247	ndhF-rpl32 IGS	SSC
c	(A)10(TA)5	20	115428	115447	rpl32 CDS	SSC
c	(T)14ctagagtaataaaagctagatttc taagtatggaaaag(T)10aaactgaac ttatgaagatacttaataaa(TAT)4	103	115559	115661	rpl32-trnL UAG IGS	SSC
c	(T)11acctttcta(T)11gagttctttata aataggttt(A)10ggtcgtttttggagag aaccgaaggtaagttttgtttccgttcccc agactgttaaaaaacaaaaattttgttttttc (T)11	151	125377	125527	ycf1 CDS	SSC
c*	(A)11(AAT)4*aaaggaaaaaattc aaataaaaacgaggagaaggaatagga agtctatttttgtaaaaggataagtacaat gggattcaagatc(A)11(AAT)4*	126	28479	28604	rpoB-trnC GCA IGS	LSC

Table S5 Percentages of variable characters in coding and noncoding regions within 20 Orchidaceae species.

Table S5a Percentages of variable sites in the introns and spacers (IGS) region.

Fragments	Length (bp)	Aligned length (bp)	Variable positions	Nucleotide substitutions	Number of indels	Total length of indels	Percent variability (%)
psbA-trnK(UUU)	317	317	212	59	10	153	39.66%
trnK(UUU)-matK	422-434	434	331	43	10	288	33.97%
matK-trnK(UUU)	1679-1723	1729	1327	250	42	1077	42.07%
trnK(UUU)-rps16	664-1089	1089	973	166	37	807	63.64%
rps16 intron	1576-1892	2246	1620	309	51	1311	36.51%
rps16-trnQ(UUG)	1394-1395	1395	1340	70	12	1270	59.85%
trnQ(UUG)-psbK	482	482	342	124	26	218	51.72%
psbK-psbI	736-791	791	692	118	28	574	59.59%
trnS(GCU)-trnG(UCC)	1641	1641	1599	80	17	1519	69.78%
trnG(UCC) intron	794-824	824	369	161	33	208	29.89%
trnR(UCU)-atpA	191-362	362	339	32	12	307	65.67%
atpF intron	1218-1293	1293	840	165	37	675	30.84%
atpF-atpH	623	623	576	39	10	537	51.04%
atpH-atpI	1127-1145	1145	1054	127	25	927	62.55%
atpI-rps2	542-628	628	541	64	14	477	47.27%
rps2-rpoC2	280-307	307	210	49	12	161	38.61%
rpoC2-rpoC1	242-254	254	153	58	9	95	39.88%
rpoC1 intron	853-887	890	482	169	28	313	32.56%
rpoB-trnC(GCA)	2011-2098	2098	1847	213	46	1634	50.78%
trnC(GCA)-petN	1115-1138	1138	1095	107	20	988	74.71%
petN-psbM	974-1202	1202	1138	156	34	982	74.80%
psbM-trnD(GUC)	1565	1565	1488	163	20	1325	70.38%
trnD(GUC)-trnY(GUA)	407	407	345	62	14	283	55.07%
trnE(UUC)-trnT(GGU)	2034	2034	1990	29	11	1961	47.62%
trnT(GGU)-psbD	1625-1626	1626	1466	112	21	1354	45.39%
psbC-trnS(UGA)	236-237	237	185	52	13	133	55.56%
trnS(UGA)-psbZ	351-352	352	229	79	12	150	42.52%
psbZ-trnG(UCC)	468	468	345	110	18	235	51.00%
trnG(UCC)-trnfM(CAU)	321-322	322	308	7	5	301	46.15%
psaA-ycf3	856-1088	1088	859	241	32	618	54.38%
ycf3 intron1	1228	1228	766	181	34	585	31.76%
ycf3 intron2	843-845	845	327	157	25	170	26.00%
ycf3-trnS(GGA)	716	716	614	37	31	577	40.00%
trnS(GGA)-rps4	429-430	430	326	64	16	262	43.48%
rps4-trnT(UGU)	595	595	478	69	20	409	43.20%
trnT(UGU)-trnL(UAA)	1516	1516	1408	181	37	1227	66.87%
trnL(UAA) intron	1147-1148	1148	882	130	28	752	37.26%
trnL(UAA)-trnF(GAA)	618	618	567	71	16	496	63.04%
trnF(GAA)-ndhJ	1037	1037	1020	25	4	995	63.04%
ndhC-trnV(UAC)	1744	1744	1694	58	15	1636	59.35%
trnV(UAC) intron	738-750	750	342	146	22	196	29.17%
trnV(UAC)-trnM(CAU)	260-263	265	156	56	13	100	38.76%
trnM(CAU)-atpE	238-241	241	171	70	13	101	54.25%

atpB-rbcL	1436-1439	1439	1164	289	58	875	55.79%
rbcL-accD	1163-1714	1714	1467	262	48	1205	55.66%
accD-psaI	604-1110	1119	1029	112	23	917	60.00%
psaI-ycf4	528-720	720	403	168	28	235	38.21%
ycf4-cemA	1080-1520	1520	1213	180	22	1033	39.69%
cemA-petA	348-484	484	266	104	20	162	36.26%
petA-psbJ	1442-1452	1452	1332	88	15	1244	46.19%
psbE-petL	1754	1754	1547	185	31	1362	51.06%
petL-petG	226	226	132	49	10	83	38.56%
trmW(CCA)-trnP(UGG)	247	247	208	51	10	157	61.00%
trnP(UGG)-psaJ	552-553	553	460	67	16	393	47.16%
psaJ-rpl33	750-818	818	712	78	17	634	47.26%
rpl33-rps18	259	259	201	51	13	201	90.14%
rps18-rpl20	240-394	394	321	54	12	267	47.48%
rpl20-rps12	1094-1124	1124	696	147	26	549	28.79%
clpP intron1	828	829	477	150	38	327	34.81%
clpP intron2	1466-1472	1473	1048	223	43	825	38.49%
clpP-psbB	1186	1186	990	136	25	854	45.10%
psbB-psbT	768-777	777	726	29	4	697	39.29%
psbH-petB	219-806	806	454	171	28	283	36.12%
petB intron	248-854	854	400	200	34	200	34.01%
petB-petD	260-267	267	179	60	15	119	46.01%
petD intron	1083-1085	1085	648	256	45	392	40.79%
petD-rpoA	16-278	278	196	54	12	142	44.59%
rpoA-rps11	223	223	178	30	8	148	45.78%
rps11-rpl36	250	250	212	60	15	152	66.37%
rpl36-infA	219-226	226	170	44	7	126	47.66%
rps8-rpl14	494-927	927	855	68	12	787	52.63%
rpl16 intron	1930-1933	1933	1486	276	49	1210	42.10%
rpl16-rps3	220-229	229	159	78	14	81	56.79%
rpl22-rps19	349-352	352	305	11	6	294	26.56%
rpl2 intron	711-715	715	95	39	6	56	6.77%
rpl2-rpl23	4-403	484	326	22	3	304	13.66%
ycf2-trnI(CAA)	943-1403	1403	830	120	25	710	20.19%
trnI(CAU)-ndhB	649-656	656	581	43	5	538	39.02%
ndhB intron	720	720	720	0	1	720	100.00%
ndhB-rps7	380	380	149	35	9	114	16.00%
rps12 intorn	472-565	573	63	43	4	20	8.44%
rps12-trnV(GAC)	2390-2393	2393	2113	53	14	2060	19.31%
trnV(GAC)-rrn16	283	283	122	47	8	75	25.46%
rrn16-trnI(GAU)	332-346	346	111	29	8	82	13.60%
trnI(GAU) intron	1076-1086	1086	330	96	18	234	13.10%
trnA(UGC) intron	856	856	136	54	15	82	8.75%
rrn4.5-rrn5	243	243	158	70	14	88	49.70%
rrn5-trnR (ACG)	301	301	277	7	2	277	34.62%
trnR (ACG)-trnN(GUU)	880	880	777	45	12	732	35.63%
trnN(GUU)-ycf1	534	534	412	86	15	326	45.29%
ndhF-rpl32	1676-1687	1687	1673	48	11	1625	80.82%
rpl32-trnL(UAG)	1472-1487	1487	1416	215	48	1201	78.74%

trnL(UAG)-ccsA	336-743	744	442	164	12	278	36.82%
ccsA-ndhD	295-439	474	474	0	1	474	100.00%
psaC-ndhE	1074	1074	979	84	22	895	52.74%
ndhE-ndhG	274-289	289	274	57	9	217	81.48%
ndhH-rps15	236-274	274	258	54	5	204	78.67%
rps15-ycf1	442-3024	3024	2206	778	59	1428	50.57%

Table S5b Percentages of variable sites in the protein coding sequences (CDS) region.

Fragments	Length (bp)	Aligned length (bp)	Variable positions	Nucleotide substitutions	Number of indels	Total length of indels	Percent variability (%)
psbA	2259	2259	245	239	1	6	10.65%
matK	1377-1611	1611	605	525	18	80	35.05%
rps16	240-351	357	253	157	11	96	61.76%
psbK	186	186	48	48	0	0	25.81%
psbI	111-156	156	59	59	0	0	37.82%
atpA	1524-1563	1563	306	303	3	3	19.58%
atpF	567-633	633	239	158	6	81	29.39%
atpH	252	252	40	34	1	6	14.17%
atpI	744-762	762	141	137	2	4	18.29%
rps2	714	714	189	171	1	18	24.68%
rpoC2	4275-4285	4287	1161	964	30	197	24.13%
rpoC1	2055-2091	2091	411	336	7	75	16.96%
rpoB	3222-3285	3285	621	606	8	15	18.73%
psbD	853-1083	1083	129	107	2	22	10.25%
psbC	1224-1422	1422	167	164	3	3	11.74%
psbZ	189	189	23	23	0	0	12.17%
rps14	369	369	71	71	0	0	19.24%
psaB	2217	2217	278	265	2	13	12.10%
psaA	2259	2259	245	239	1	6	10.65%
ycf3	504-621	621	187	187	0	0	30.11%
rps4	606	606	112	112	0	0	18.48%
ndhJ	477-486	486	55	55	0	0	11.32%
ndhK	583-915	915	743	189	13	554	54.01%
ndhC	240-363	363	284	116	5	168	60.50%
atpE	405	405	84	81	3	3	20.74%
atpB	1509-1536	1536	268	235	3	33	15.80%
rbcL	1452-1488	1488	243	226	2	17	15.48%
accD	3234-3264	3264	2399	548	55	1851	41.08%
ycf4	282-567	567	133	118	4	15	21.94%
cemA	216-690	690	226	167	7	59	27.27%
petA	978	978	162	144	5	18	15.44%
psbE	252	252	28	28	0	0	11.11%
psaJ	129-159	159	32	31	1	1	20.13%
rpl33	225	225	68	44	1	24	22.28%
rps18	444	444	218	80	6	138	27.56%
rpl20	354-420	420	144	137	2	7	33.49%
rps12	379-393	393	266	52	5	214	30.98%
clpP	594-675	675	256	236	4	20	36.42%

psbB	1471-1527	1527	241	238	3	3	15.78%
petB	459-700	726	161	157	2	4	21.96%
petD	483-669	711	235	229	1	6	32.58%
rpoA	1020	1020	282	225	7	57	23.92%
rps11	435	435	127	107	4	20	26.49%
infA	246-255	255	76	55	2	21	24.15%
rps8	396	396	87	86	1	1	21.97%
rpl14	369	369	71	71	0	0	19.24%
rpl16	360-435	435	128	113	4	15	27.59%
rps3	672-678	678	199	171	5	28	26.87%
rpl22	372-552	552	276	241	5	35	47.13%
rps19	210-279	279	40	40	0	0	14.34%
rpl2	738-825	825	66	63	1	3	7.78%
rpl23	292-303	303	52	30	2	22	11.31%
ycf2	6037-7587	7587	3105	885	67	2220	17.52%
ndhB	1542	1542	112	83	5	29	5.80%
rps7	474	474	45	39	1	6	8.53%
ndhF	138-2268	2268	1664	639	27	1025	52.44%
rpl32	152-174	174	120	70	6	50	58.46%
ccsA	607-1014	1014	375	261	14	114	30.09%
ndhD	963-1560	1560	665	190	6	475	17.97%
psaC	246	246	31	31	0	0	12.60%
ndhE	306-321	321	70	70	0	0	21.81%
ndhG	258-531	531	66	66	0	0	12.43%
ndhI	510-546	561	424	175	7	249	57.05%
ndhA	1107	1107	661	105	4	556	19.64%
ndhH	1182	1182	116	116	0	0	9.81%
rps15	240-276	276	99	86	3	13	33.46%
ycf1	4929-6582	6582	5030	1299	117	3731	47.71%

Table S6. Maximum likelihood parameter estimates for the 68 genes of Orchidaceae species.

gene	model	l	np	Estimates of parameters		positively selected sites				
accD	model0	-4517.320743	39	omega (dN/dS) =0.89282						
	model1	-4442.667189	40	p=0.46134 0.53866	w=0.09518 1.00000					
	model2	-4400.090826	42	p=0.33185 0.54617 0.12198	w=0.06940 1.00000 5.13144	2K0.991**	57N1.000**	120R0.999**	131K0.999**	134E0.999**
						135S1.000**	197T0.998**	203G0.994**		
	model3	-4398.337394	43	p=0.49953 0.42750 0.07297	w=0.19748 1.61254 6.94809					
	model7	-4446.04334	40	p =0.03115 q =0.01766						
	model8	-4400.450869	42	p0 =0.87303 p =0.11896 q =0.06556	(p1 =0.12697) w =5.08006	2K0.997**	48D0.991**	50S0.996**	57N1.000**	120R0.999**
						131K1.000**	134E1.000**	135S1.000**	197T0.999**	203G0.997**
atpA	model0	-4424.930851	39	omega (dN/dS) =0.11670						
	model1	-4347.474492	40	p=0.89462 0.10538	w=0.03181 1.00000					
	model2	-4345.645133	42	p=0.90348 0.07695 0.01957	w=0.03602 1.00000 2.37806					
	model3	-4345.366717	43	p=0.87765 0.08806 0.03430	w=0.02995 0.63737 2.10262					
	model7	-4350.946376	40	p =0.06556 q =0.42200						
	model8	-4345.671334	42	p0 =0.95375 p =0.17844 q =2.19383	(p1 =0.04625) w =1.87963					
atpB	model0	-3948.210705	39	omega (dN/dS) =0.11709						
	model1	-3911.611259	40	p=0.91395 0.08605	w=0.04033 1.00000					
	model2	-3911.441903	42	p=0.91651 0.08031 0.00319	w=0.04174 1.00000 2.76638					
	model3	-3911.387419	43	p=0.66323 0.29442 0.04235	w=0.00000 0.22274 1.38618					
	model7	-3912.749945	40	p =0.08886 q =0.63014						
	model8	-3911.313303	42	p0 =0.97945 p =0.15574 q =1.49864	(p1 =0.02055) w =1.79864					
atpE	model0	-1176.94085	39	omega (dN/dS) =0.30034						
	model1	-1161.337278	40	p=0.78444 0.21556	w=0.09001 1.00000					
	model2	-1159.441353	42	p=0.82220 0.14346 0.03434	w=0.11640 1.00000 3.88675					
	model3	-1159.370023	43	p=0.85437 0.12304 0.02259	p=0.85437 0.12304 0.02259					
	model7	-1162.710609	40	p =0.16506 q =0.38008						
	model8	-1159.644113	42	p0 =0.90746 p =1.49732 q =7.45995	(p1 =0.09254) w =2.22593					
atpF	model0	-1961.396306	39	omega (dN/dS) =0.33560						
	model1	-1942.201957	40	p=0.73478 0.26522	w=0.13115 1.00000					
	model2	-1941.616819	42	p=0.80587 0.03832 0.15582	w=0.16731 1.00000 1.43596					
	model3	-1940.963643	43	p=0.24942 0.61957 0.13102	w=0.00000 0.29954 1.62466					
	model7	-1943.365353	40	p =0.33278 q =0.59794						

	model8	-1941.208783	42	p0 =0.87164 p =1.11301 q =3.89127	(p1 =0.12836) w =1.60799		
atpH	model0	-542.02404	39	omega (dN/dS) =0.06818			
	model1	-542.006769	40	p=0.99358 0.00642	w=0.06406 1.00000		
	model2	-542.006769	42	p=0.99358 0.00210 0.00432	w=0.06406 1.00000 1.00000		
	model3	-541.867784	43	p=0.43689 0.20553 0.35758	w=0.00000 0.12140 0.12140		
	model7	-541.875402	40	p =0.85973 q =11.32282			
	model8	-541.875499	42	p0 =0.99999 p =0.86009 q =11.32854	(p1 =0.00001) w =1.00000		
atpI	model0	-2043.804493	39	omega (dN/dS) =0.15030			
	model1	-2022.584987	40	p=0.87209 0.12791	w=0.04412 1.00000		
	model2	-2022.219882	42	p=0.88401 0.09904 0.01695	w=0.04978 1.00000 2.43569		
	model3	-2021.970185	43	p=0.62352 0.32701 0.04947	w=0.00000 0.27198 1.92187		
	model7	-2023.321405	40	p =0.09837 q =0.50540			
	model8	-2021.985587	42	p0 =0.95705 p =0.22592 q =1.90193	(p1 =0.04295) w =2.01657		
ccsA	model0	-1118.278228	39	omega (dN/dS) =0.81855			
	model1	-1105.658405	40	p=0.52745 0.47255	w=0.10558 1.00000		
	model2	-1096.541843	42	p=0.64140 0.20081 0.15779	w=0.25967 1.00000 3.83925		
	model3	-1096.53864	43	p=0.68737 0.16492 0.14770	p=0.68737 0.16492 0.14770		
	model7	-1106.758699	40	p =0.12010 q =0.09121			
	model8	-1096.549564	42	p0 =0.82609 p =1.41334 q =2.11415	(p1 =0.17391) w =3.68361	43I0.995**	70A0.996**
cemA	model0	-591.29386	39	omega (dN/dS) =0.34563			
	model1	-588.007972	40	p=0.76281 0.23719	p=0.76281 0.23719		
	model2	-587.633254	42	p=0.84091 0.00000 0.15909	w=0.22296 1.00000 1.59616		
	model3	-587.633254	43	p=0.26097 0.57994 0.15909	w=0.22296 0.22296 1.59616		
	model7	-588.928797	40	p =0.68781 q =1.12286			
	model8	-587.654195	42	p0 =0.84292 p =28.88357 q =99.00000	(p1 =0.15708) w =1.60416		
clpP	model0	-1947.160568	39	omega (dN/dS) =0.39134			
	model1	-1935.069689	40	p=0.61510 0.38490	w=0.13197 1.00000		
	model2	-1935.069689	42	p=0.61510 0.26952 0.11539	w=0.13197 1.00000 1.00000		
	model3	-1933.282794	43	p=0.32013 0.64934 0.03054	w=0.00000 0.57211 2.95424		
	model7	-1934.73402	40	p =0.34032 q =0.43015			
	model8	-1933.741944	42	p0 =0.98289 p =0.44013 q =0.61467	(p1 =0.01711) w =3.54442		
infA	model0	-660.388734	39	omega (dN/dS) =0.11934			
	model1	-660.024396	40	p=0.95822 0.04178	w=0.10112 1.00000		

	model2	-660.024396	42	p=0.95822 0.02137 0.02041	w=0.10112 1.00000 1.00000	
	model3	-659.777366	43	p=0.23596 0.25996 0.50408	w=0.02433 0.02433 0.22977	
	model7	-659.783332	40	p =0.87556 q =5.75383		
	model8	-659.783387	42	p0 =0.99999 p =0.87567 q =5.75488	(p1 =0.00001) w =1.00000	
matK	model0	-5715.255186	39	omega (dN/dS) =0.44157		
	model1	-5650.370591	40	p=0.66246 0.33754	w=0.16225 1.00000	
	model2	-5644.060931	42	p=0.66531 0.29526 0.03943	w=0.18473 1.00000 2.84240	
	model3	-5641.74757	43	p=0.40886 0.51793 0.07321	w=0.08281 0.57798 2.35733	
	model7	-5654.260871	40	p =0.43664 q =0.57283		
	model8	-5641.988921	42	p0 =0.93554 p =0.73363 q =1.22112	(p1 =0.06446) w =2.43593	
petA	model0	-2609.741206	39	omega (dN/dS) =0.16156		
	model1	-2575.421129	40	p=0.87182 0.12818	w=0.04623 1.00000	
	model2	-2573.940165	42	p=0.88598 0.09457 0.01945	w=0.05444 1.00000 2.96782	
	model3	-2572.933212	43	p=0.62521 0.33659 0.03820	w=0.00000 0.30240 2.51099	
	model7	-2576.796892	40	p =0.09702 q =0.48253		
	model8	-2573.121887	42	p0 =0.96389 p =0.24226 q =1.87986	(p1 =0.03611) w =2.53368	
petB	model0	-1080.581644	39	omega (dN/dS) =0.08863		
	model1	-1067.561634	40	p=0.90048 0.09952	w=0.00716 1.00000	
	model2	-1067.561636	42	p=0.90048 0.04215 0.05736	w=0.00716 1.00000 1.00000	
	model3	-1067.492398	43	p=0.87308 0.11211 0.01481	w=0.00000 0.71134 1.44179	
	model7	-1067.522296	40	p =0.02716 q =0.25678		
	model8	-1067.492826	42	p0 =0.96957 p =0.01283 q =0.20694	(p1 =0.03043) w =1.19628	
petD	model0	-1330.950652	39	omega (dN/dS) =0.08795		
	model1	-1286.808743	40	p=0.93602 0.06398	w=0.00169 1.00000	
	model2	-1284.924274	42	p=0.94033 0.03636 0.02331	w=0.00245 1.00000 2.55005	
	model3	-1285.172258	43	p=0.15820 0.78665 0.05515	w=0.00319 0.00332 1.65639	
	model7	-1287.764137	40	p =0.00500 q =0.03478		
	model8	-1285.172989	42	p0 =0.94498 p =0.34712 q =93.92009	(p1 =0.05502) w =1.65918	2G0.991**
petG	model0	-305.266194	39	omega (dN/dS) =0.08873		
	model1	-299.472955	40	p=0.92418 0.07582	w=0.01304 1.00000	
	model2	-266.875911	42	p=0.81068 0.15996 0.02936	w=0.00000 1.00000 94.34796	
	model3	-265.49392	43	p=0.90875 0.06315 0.02809	w=0.04458 4.11622 117.37218	
	model7	-299.596622	40	p =0.00500 q =0.03082		

	model8	-267.08922	42	p0 =0.96849 p =0.01116 q =0.07239	(p1 =0.03151) w =83.05482	
petL	model0	-242.629156	39	omega (dN/dS) =1.96980		
	model1	-243.012755	40	p=0.99999 0.00001	w=1.00000 1.00000	
	model2	-242.37431	42	p=0.80360 0.08016 0.11624	w=1.00000 1.00000 3.78355	
	model3	-241.539084	43	p=0.11603 0.81411 0.06986	w=0.08045 2.12488 6.98690	
	model7	-242.786121	40	p =33.01305 q =0.00500		
	model8	-241.411408	42	p0 =0.14093 p =11.16185 q =99.00000	(p1 =0.85907) w =2.55120	16S0.992**
petN	model0	-237.275342	39	omega (dN/dS) =0.27583		
	model1	-236.936612	40	p=0.65920 0.34080	w=0.19770 1.00000	
	model2	-236.936612	42	p=0.65920 0.24698 0.09382	w=0.19770 1.00000 1.00000	
	model3	-236.214357	43	p=0.31796 0.60837 0.07367	w=0.06122 0.41992 0.41992	
	model7	-236.420972	40	p =1.27564 q =2.76467		
	model8	-236.420982	42	p0 =0.99999 p =1.27565 q =2.76472	(p1 =0.00001) w =1.00000	
psaA	model0	-5136.762038	39	omega (dN/dS) =0.05780		
	model1	-5111.716694	40	p=0.96448 0.03552	w=0.02466 1.00000	
	model2	-5111.488393	42	p=0.97342 0.00000 0.02658	w=0.02799 1.00000 1.29777	
	model3	-5111.468399	43	p=0.58007 0.39782 0.02210	w=0.00000 0.07705 1.40048	
	model7	-5113.821116	40	p =0.04556 q =0.64081		
	model8	-5111.472168	42	p0 =0.97751 p =0.51170 q =15.11825	(p1 =0.02249) w =1.38945	
psaB	model0	-5170.307983	39	omega (dN/dS) =0.04680		
	model1	-5155.049981	40	p=0.97035 0.02965	w=0.02410 1.00000	
	model2	-5155.049983	42	p=0.97035 0.02965 0.00000	w=0.02410 1.00000 27.56975	
	model3	-5154.11512	43	p=0.82158 0.16466 0.01376	w=0.00000 0.22239 0.94426	
	model7	-5154.313684	40	p =0.06077 q =1.01864		
	model8	-5154.128829	42	p0 =0.98958 p =0.08166 q =1.68835	(p1 =0.01042) w =1.00000	
psaC	model0	-608.425433	39	omega (dN/dS) =0.00374		
	model1	-608.425799	40	p=0.99999 0.00001	w=0.00374 1.00000	
	model2	-586.565724	42	p=0.98779 0.00000 0.01221	w=0.00552 1.00000 22.99982	
	model3	-608.425433	43	p=0.18748 0.39725 0.41527	w=0.00368 0.00375 0.00376	
	model7	-608.426036	40	p =0.40565 q =99.00000		
	model8	-586.566036	42	p0 =0.98779 p =0.58577 q =99.00000	(p1 =0.01221) w =23.00686	
psaI	model0	-320.129977	39	omega (dN/dS) =0.23679		
	model1	-318.714876	40	p=0.86381 0.13619	w=0.15130 1.00000	

	model2	-318.572337	42	p=0.90505 0.00000 0.09495	w=0.16916 1.00000 1.53648
	model3	-318.572337	43	p=0.27551 0.62954 0.09495	w=0.16916 0.16916 1.53648
	model7	-319.324893	40	p =0.83859 q =2.31953	
	model8	-318.5934	42	p0 =0.90700 p =20.55635 q =99.00000	(p1 =0.09300) w =1.54699
psaJ	model0	-359.746551	39	omega (dN/dS) =0.14613	
	model1	-358.46201	40	p=0.87158 0.12842	w=0.05538 1.00000
	model2	-358.46201	42	p=0.87158 0.06648 0.06194	w=0.05538 1.00000 1.00000
	model3	-358.081173	43	p=0.39829 0.26038 0.34133	w=0.00000 0.00000 0.43577
	model7	-358.187425	40	p =0.19523 q =1.06158	
	model8	-358.187431	42	p0 =0.99999 p =0.19523 q =1.06166	(p1 =0.00001) w =1.00000
psbA	model0	-2511.403818	39	omega (dN/dS) =0.03212	
	model1	-2491.668779	40	p=0.97524 0.02476	w=0.00968 1.00000
	model2	-2491.668779	42	p=0.97524 0.02476 0.00000	w=0.00968 1.00000 25.04824
	model3	-2491.42154	43	p=0.88997 0.09907 0.01096	w=0.00000 0.17852 1.56221
	model7	-2494.443024	40	p =0.01122 q =0.22453	
	model8	-2491.421612	42	p0 =0.98904 p =0.01573 q =0.44677	(p1 =0.01096) w =1.56208
psbB	model0	-3808.899355	39	-3808.899355	
	model1	-3766.3505	40	p=0.95422 0.04578	w=0.01989 1.00000
	model2	-3763.846243	42	p=0.95639 0.04128 0.00232	w=0.02102 1.00000 9.02702
	model3	-3764.021172	43	p=0.79392 0.18908 0.01700	w=0.00000 0.19149 2.19264
	model7	-3769.386058	40	p =0.03979 q =0.48101	
	model8	-3764.05558	42	p0 =0.98462 p =0.09903 q =2.11446	(p1 =0.01538) w =2.30566
psbC	model0	-2827.930076	39	omega (dN/dS) =0.02969	
	model1	-2820.767621	40	p=0.96855 0.03145	w=0.00803 1.00000
	model2	-2820.767621	42	p=0.96855 0.03145 0.00000	w=0.00803 1.00000 11.85812
	model3	-2819.766744	43	p=0.40229 0.52736 0.07035	w=0.00000 0.00000 0.47444
	model7	-2820.105288	40	p =0.01160 q =0.24552	
	model8	-2820.105384	42	p0 =0.99999 p =0.01124 q =0.23749	(p1 =0.00001) w =1.00000
psbD	model0	-1815.134795	39	omega (dN/dS) =0.05078	
	model1	-1800.167919	40	p=0.95451 0.04549	w=0.00919 1.00000
	model2	-1800.049079	42	p=0.96376 0.00000 0.03624	w=0.01205 1.00000 1.29753
	model3	-1800.049079	43	p=0.06195 0.90181 0.03624	p=0.06195 0.90181 0.03624
	model7	-1801.114182	40	p =0.01071 q =0.18463	

	model8	-1800.054499	42	p0 =0.96400 p =1.26244 q =99.00000	(p1 =0.03600) w =1.30012	
psbE	model0	-599.291915	39	omega (dN/dS) =0.04113		
	model1	-598.461381	40	p=0.97567 0.02433	w=0.02703 1.00000	
	model2	-571.80637	42	p=0.93462 0.05345 0.01193	w=0.03632 1.00000 35.32003	
	model3	-598.322901	43	p=0.22818 0.63988 0.13194	w=0.00906 0.00908 0.28357	
	model7	-598.32458	40	p =0.12084 q =2.25751		
	model8	-571.805219	42	p0 =0.98807 p =0.09215 q =0.94217	(p1 =0.01193) w =35.16835	
petF	model0	-277.358932	39	omega (dN/dS) =0.04208		
	model1	-271.462892	40	p=0.96537 0.03463	w=0.00000 1.00000	
	model2	-230.437805	42	p=0.90788 0.06710 0.02502	w=0.00000 1.00000 832.51000	
	model3	-240.672727	43	p=0.94993 0.00000 0.05007	w=0.00000 0.00000 234.98351	
	model7	-272.329411	40	p =0.00809 q =0.12289		
	model8	-230.514018	42	p0 =0.97498 p =0.00500 q =0.04833	(p1 =0.02502) w =866.26030	
psbH	model0	-740.932966	39	omega (dN/dS) =0.17586		
	model1	-720.475517	40	p=0.82386 0.17614	w=0.03513 1.00000	
	model2	-691.717304	42	p=0.75222 0.20905 0.03873	w=0.03633 1.00000 18.77007	
	model3	-691.356216	43	p=0.79523 0.16869 0.03607	w=0.05536 1.43491 21.13097	
	model7	-720.650379	40	p =0.09900 q =0.43890		
	model8	-691.906156	42	p0 =0.96080 p =0.06800 q =0.21099	(p1 =0.03920) w =18.59309	18D0.992**
psbI	model0	-239.43191	39	omega (dN/dS) =0.01840		
	model1	-239.432097	40	p=0.99999 0.00001	w=0.01840 1.00000	
	model2	-239.43191	42	p=1.00000 0.00000 0.00000	w=0.01840 1.00000 56.32581	
	model3	-239.43191	43	p=0.20929 0.38381 0.40690	w=0.01840 0.01840 0.01840	
	model7	-239.439751	40	p =1.90541 q =99.00000		
	model8	-239.440106	42	p0 =0.99999 p =1.90527 q =99.00000	(p1 =0.00001) w =70.49267	
psbJ	model0	-353.697761	39	omega (dN/dS) =0.06517		
	model1	-351.952162	40	p=0.95851 0.04149	w=0.03587 1.00000	
	model2	-328.411838	42	p=0.89100 0.08459 0.02441	w=0.05871 1.00000 32.07455	
	model3	-351.74862	43	p=0.09916 0.83334 0.06750	w=0.03033 0.03033 0.58238	
	model7	-351.976214	40	p =0.16949 q =2.13609		
	model8	-328.652343	42	p0 =0.97559 p =0.13983 q =0.81750	(p1 =0.02441) w =32.22586	
psbK	model0	-766.990787	39	omega (dN/dS) =0.26314		
	model1	-760.012062	40	p=0.75557 0.24443	w=0.11095 1.00000	

	model2	-744.164799	42	p=0.63605 0.34773 0.01621	w=0.12313 1.00000 15.11618
	model3	-759.756546	43	p=0.42866 0.25665 0.31469	w=0.08231 0.08231 0.68368
	model7	-760.119435	40	p =0.47063 q =1.26450	
	model8	-745.437735	42	p0 =0.97218 p =0.35306 q =0.43787	(p1 =0.02782) w =10.11004
psbL	model0	-288.614063	39	omega (dN/dS) =0.03407	
	model1	-286.489312	40	p=0.96392 0.03608	p=0.96392 0.03608
	model2	-259.767919	42	p=0.94861 0.00000 0.05139	w=0.05010 1.00000 38.77087
	model3	-259.767918	43	p=0.36916 0.57945 0.05139	w=0.05010 0.05010 38.77082
	model7	-286.674553	40	p =0.00956 q =0.18825	
	model8	-259.797649	42	p0 =0.94859 p =5.25355 q =99.00000	(p1 =0.05141) w =37.24594
psbM	model0	-334.969904	39	omega (dN/dS) =0.12830	
	model1	-331.359543	40	p=0.90781 0.09219	w=0.05764 1.00000
	model2	-301.85983	42	p=0.72888 0.24251 0.02861	w=0.07813 1.00000 42.15404
	model3	-300.854552	43	p=0.88218 0.08920 0.02861	w=0.16238 3.02099 48.78520
	model7	-331.845708	40	p =0.19378 q =1.15562	
	model8	-302.066382	42	p0 =0.97138 p =0.02157 q =0.04217	(p1 =0.02862) w =43.90437
psbN	model0	-359.736673	39	omega (dN/dS) =0.10192	
	model1	-359.435571	40	p=0.96774 0.03226	w=0.08030 1.00000
	model2	-313.12015	42	p=0.73752 0.23973 0.02275	w=0.17457 1.00000 209.49256
	model3	-312.359168	43	p=0.95638 0.02088 0.02275	w=0.29131 17.62527 234.31587
	model7	-359.559478	40	p =0.77078 q =6.38081	
	model8	-313.147882	42	p0 =0.97725 p =0.35680 q =0.57727	(p1 =0.02275) w =211.19032
psbT	model0	-284.919434	39	omega (dN/dS) =0.05013	
	model1	-284.919585	40	p=0.99999 0.00001	w=0.05013 1.00000
	model2	-284.919434	42	p=1.00000 0.00000 0.00000	w=0.05013 1.00000 1.00000
	model3	-284.919446	43	p=0.25520 0.24043 0.50437	w=0.05013 0.05013 0.05013
	model7	-284.949936	40	p =5.34652 q =99.00000	
	model8	-284.950262	42	p0 =0.99999 p =5.34652 q =99.00000	(p1 =0.00001) w =55.66033
psbZ	model0	-475.830764	39	omega (dN/dS) =0.09533	
	model1	-475.691511	40	p=0.96726 0.03274	w=0.07869 1.00000
	model2	-447.25251	42	p=0.77241 0.21168 0.01591	w=0.06036 1.00000 47.24918
	model3	-474.909469	43	p=0.60199 0.16757 0.23044	w=0.00000 0.26053 0.26053
	model7	-475.058128	40	p =0.36313 q =3.02296	

	model8	-447.137387	42	p0 =0.98409 p =0.17568 q =0.54878	(p1 =0.01591) w =45.83413						
rbcL	model0	-3911.862654	39	omega (dN/dS) =0.12988							
	model1	-3817.092508	40	p=0.92902 0.07098	w=0.02955 1.00000						
	model2	-3796.588213	42	p=0.94363 0.03703 0.01934	w=0.03999 1.00000 4.92488	89P0.992**	251M0.997**	375L0.999**	461I0.999**		
	model3	-3796.373581	43	p=0.92228 0.05702 0.02071	w=0.03366 0.67761 4.70803						
	model7	-3820.894279	40	p =0.01878 q =0.12573							
	model8	-3796.826963	42	p0 =0.97832 p =0.13976 q =1.72648	(p1 =0.02168) w =4.55273	89P0.999**	225L0.997**	251M1.000**	375L1.000**	449S0.998**	
						461I1.000**	470E0.991**				
rpl2	model0	-1326.121554	39	omega (dN/dS) =0.32434							
	model1	-1324.343079	40	p=0.66295 0.33705	w=0.00000 1.00000						
	model2	-1324.323355	42	p=0.68983 0.00000 0.31017	w=0.00000 1.00000 1.12375						
	model3	-1324.323355	43	p=0.68983 0.19515 0.11502	w=0.00000 1.12376 1.12376						
	model7	-1324.378166	40	p =0.01443 q =0.02793							
	model8	-1324.323355	42	p0 =0.68983 p =0.00500 q =2.78679	(p1 =0.31017) w =1.12376						
rpl14	model0	-1160.017074	39	omega (dN/dS) =0.14354							
	model1	-1156.500076	40	p=0.91664 0.08336	w=0.09427 1.00000						
	model2	-1125.587083	42	p=0.86592 0.12594 0.00814	w=0.11014 1.00000 29.31055						
	model3	-1156.077922	43	p=0.55420 0.27588 0.16992	w=0.07013 0.07013 0.57093						
	model7	-1156.265182	40	p =0.46918 q =2.52882							
	model8	-1125.832751	42	p0 =0.99186 p =0.40394 q =1.51157	(p1 =0.00814) w =28.80370						
rpl16	model0	-1166.181319	39	omega (dN/dS) =0.22765							
	model1	-1152.63794	40	p=0.75481 0.24519	w=0.06140 1.00000						
	model2	-1152.63794	42	p=0.75481 0.15137 0.09381	w=0.06140 1.00000 1.00000						
	model3	-1150.689	43	p=0.54880 0.43517 0.01602	w=0.00000 0.49891 2.48991						
	model7	-1151.76835	40	p =0.18711 q =0.54406							
	model8	-1151.370521	42	p0 =0.98756 p =0.23197 q =0.74708	(p1 =0.01244) w =2.50822						
rpl20	model0	-1241.165958	39	omega (dN/dS) =0.31696							
	model1	-1223.202311	40	p=0.74624 0.25376	w=0.09418 1.00000						
	model2	-1222.567262	42	p=0.79281 0.00000 0.20719	w=0.11762 1.00000 1.36372						
	model3	-1222.567262	43	p=0.34385 0.44896 0.20719	w=0.11762 0.11762 1.36372						
	model7	-1225.165144	40	p =0.22964 q =0.47489							
	model8	-1222.595899	42	p0 =0.79489 p =13.48409 q =99.00000	(p1 =0.20511) w =1.37053						
rpl22	model0	-1287.298274	39	omega (dN/dS) =0.32659							

	model1	-1254.076303	40	p=0.69532 0.30468	w=0.06071 1.00000		
	model2	-1246.80774	42	p=0.67704 0.30263 0.02033	w=0.06164 1.00000 8.44802	47R0.995**	
	model3	-1246.115699	43	p=0.55487 0.40461 0.04052	w=0.02045 0.62303 4.59829		
	model7	-1254.310885	40	p =0.15515 q =0.32779			
	model8	-1246.283957	42	p0 =0.96782 p =0.20476 q =0.48079	(p1 =0.03218) w =5.39123	47R0.998**	100P0.996**
rpl23	model0	-494.807769	39	omega (dN/dS) =0.41070			
	model1	-494.033568	40	p=0.58808 0.41192	w=0.00000 1.00000		
	model2	-494.031094	42	p=0.60191 0.00000 0.39809	w=0.00000 1.00000 1.05503		
	model3	-494.031094	43	p=0.37287 0.22904 0.39809	w=0.00000 0.00000 1.05503		
	model7	-494.036598	40	p =0.00500 q =0.00754			
	model8	-494.031094	42	p0 =0.60191 p =0.00500 q =1.81908	(p1 =0.39809) w =1.05503		
rpl32	model0	-574.150687	39	omega (dN/dS) =0.27924			
	model1	-557.620919	40	p=0.65562 0.34438	w=0.02683 1.00000		
	model2	-549.971425	42	p=0.65022 0.33000 0.01978	w=0.03908 1.00000 16.81869	38R1.000**	
	model3	-555.460161	43	p=0.11688 0.83320 0.04992	w=0.00000 0.25206 7.36254		
	model7	-559.633994	40	p =0.35427 q =0.87685			
	model8	-551.135135	42	p0 =0.98160 p =0.43722 q =1.16247	(p1 =0.01840) w =14.44038	38R1.000**	
rpl33	model0	-654.804015	39	omega (dN/dS) =0.28454			
	model1	-632.987526	40	p=0.73620 0.26380	w=0.03445 1.00000		
	model2	-631.2325	42	p=0.76369 0.10207 0.13425	w=0.05493 1.00000 2.27986		
	model3	-631.176337	43	p=0.70792 0.14079 0.15129	w=0.04085 0.55749 2.25298		
	model7	-633.748894	40	p =0.01849 q =0.03720			
	model8	-631.199413	42	p0 =0.84236 p =0.28004 q =2.04463	(p1 =0.15764) w =2.21594		
rpl36	model0	-435.286609	39	omega (dN/dS) =0.06054			
	model1	-432.188161	40	p=0.86518 0.13482	w=0.02539 1.00000		
	model2	-406.83351	42	p=0.71537 0.25828 0.02634	w=0.00000 1.00000 28.47280		
	model3	-429.580303	43	p=0.37416 0.34901 0.27683	w=0.00000 0.00000 0.35000		
	model7	-430.244936	40	p =0.13307 q =1.21496			
	model8	-406.64795	42	p0 =0.97365 p =0.01436 q =0.04342	(p1 =0.02635) w =27.54440		
rpoA	model0	-3238.905022	39	omega (dN/dS) =0.36366			
	model1	-3186.355309	40	p=0.73423 0.26577	w=0.08998 1.00000		
	model2	-3180.555846	42	p=0.74782 0.20897 0.04320	w=0.10913 1.00000 3.12566		
	model3	-3179.500582	43	p=0.80118 0.18998 0.00884	w=0.13168 1.48909 6.11408		

	model7	-3190.551118	40	p =0.17186 q =0.32525					
	model8	-3181.128163	42	p0 =0.83328 p =4.35886 q =24.40070	(p1 =0.16672) w =1.82211	236L0.994**			
rpoB	model0	-8985.146872	39	omega (dN/dS) =0.20515					
	model1	-8880.915881	40	p=0.83258 0.16742	w=0.05748 1.00000				
	model2	-8874.355087	42	p=0.84800 0.13437 0.01763	w=0.06884 1.00000 3.44392				
	model3	-8870.921091	43	p=0.59644 0.37128 0.03228	w=0.00000 0.40924 2.87292				
	model7	-8883.857634	40	p =0.11531 q =0.42473					
	model8	-8871.645097	42	p0 =0.97248 p =0.23656 q =1.16204	(p1 =0.02752) w =3.02377				
rpoC1	model0	-5925.893932	39	omega (dN/dS) =0.22096					
	model1	-5844.659579	40	p=0.85827 0.14173	w=0.07246 1.00000				
	model2	-5830.082971	42	p=0.86416 0.12372 0.01212	w=0.08219 1.00000 5.47276	562D0.995**	631R0.992**		
	model3	-5830.069489	43	p=0.85726 0.13017 0.01257	w=0.07970 0.95260 5.37621				
	model7	-5851.018881	40	p =0.14430 q =0.53378					
	model8	-5831.294417	42	p0 =0.98364 p =0.25457 q =1.11796	(p1 =0.01636) w =4.70743	21R0.992**	257R0.997**	562D0.998**	631R0.998**
rpoC2	model0	-13663.46407	39	omega (dN/dS) =0.35512					
	model1	-13459.09914	40	p=0.72954 0.27046	w=0.08770 1.00000				
	model2	-13433.01015	42	p=0.75077 0.20340 0.04583	w=0.11273 1.00000 3.23586	394L0.997**			
	model3	-13432.15178	43	p=0.51159 0.40434 0.08407	w=0.04004 0.47613 2.57734				
	model7	-13471.71836	40	p =0.16660 q =0.32061					
	model8	-13432.07165	42	p0 =0.92651 p =0.44143 q =1.31625	(p1 =0.07349) w =2.71493	394L1.000**	907I0.993**		
rps2	model0	-2148.16273	39	omega (dN/dS) =0.20951					
	model1	-2132.33294	40	p=0.84551 0.15449	w=0.10182 1.00000				
	model2	-2132.332945	42	p=0.84551 0.09381 0.06067	w=0.10182 1.00000 1.00000				
	model3	-2132.332716	43	p=0.19568 0.64871 0.15561	w=0.10139 0.10139 0.99335				
	model7	-2133.552733	40	p =0.32266 q =1.06033					
	model8	-2132.341399	42	p0 =0.84902 p =11.53067 q =99.00000	(p1 =0.15098) w =1.00671				
rps3	model0	-2103.247179	39	omega (dN/dS) =0.20155					
	model1	-2086.215942	40	p=0.89397 0.10603	w=0.11965 1.00000				
	model2	-2085.520529	42	p=0.92960 0.00000 0.07040	w=0.13606 1.00000 1.48914				
	model3	-2085.312336	43	p=0.15137 0.78625 0.06238	w=0.00000 0.16971 1.58325				
	model7	-2089.653794	40	p =0.45662 q =1.62197					
	model8	-2085.408318	42	p0 =0.93734 p =3.50463 q =20.92699	(p1 =0.06266) w =1.57222				
rps4	model0	-1751.027691	39	omega (dN/dS) =0.17361					

	model1	-1740.507753	40	p=0.93787 0.06213	w=0.11247 1.00000	
	model2	-1710.82983	42	p=0.91077 0.08426 0.00496	w=0.13251 1.00000 27.40559	
	model3	-1707.960396	43	p=0.96688 0.02816 0.00496	w=0.16494 2.95427 29.30599	
	model7	-1744.070157	40	p =0.42946 q =1.86247		
	model8	-1713.636801	42	p0 =0.99503 p =0.39853 q =1.32299	(p1 =0.00497) w =28.50625	
rps7	model0	-855.59567	39	omega (dN/dS) =0.60542		
	model1	-854.305709	40	p=0.44164 0.55836	w=0.00000 1.00000	
	model2	-852.446124	42	p=0.96945 0.00000 0.03055	w=0.49238 1.00000 13.97646	
	model3	-852.446179	43	p=0.83810 0.13135 0.03055	w=0.49238 0.49239 13.97635	
	model7	-854.343458	40	p =0.00748 q =0.00501		
	model8	-852.447822	42	p0 =0.96950 p =96.27947 q =99.00000	(p1 =0.03050) w =13.99467	
rps8	model0	-1346.963062	39	omega (dN/dS) =0.17467		
	model1	-1330.459725	40	p=0.86110 0.13890	w=0.07875 1.00000	
	model2	-1304.019553	42	p=0.81438 0.17804 0.00758	w=0.08446 1.00000 20.80704	
	model3	-1303.876714	43	p=0.83874 0.15368 0.00758	w=0.09509 1.18435 21.41344	
	model7	-1331.391857	40	p =0.25664 q =1.04816		
	model8	-1305.335157	42	p0 =0.99241 p =0.22429 q =0.68848	(p1 =0.00759) w =20.55281	
rps11	model0	-1277.146802	39	omega (dN/dS) =0.13837		
	model1	-1277.147036	40	p=0.99999 0.00001	w=0.13837 1.00000	
	model2	-1277.146802	42	p=1.00000 0.00000 0.00000	w=0.13837 1.00000 1.00000	
	model3	-1275.733146	43	p=0.31824 0.27117 0.41059	w=0.00000 0.21113 0.21113	
	model7	-1276.264646	40	p =1.35474 q =7.92066		
	model8	-1276.264976	42	p0 =0.99999 p =1.35476 q =7.92090	(p1 =0.00001) w =1.00000	
rps12	model0	-458.976296	39	omega (dN/dS) =1.06338		
	model1	-454.777522	40	p=0.41162 0.58838	w=0.00000 1.00000	
	model2	-445.469994	42	p=0.35351 0.62791 0.01858	w=0.00000 1.00000 266.74739	54V0.995**
	model3	-443.356035	43	p=0.47830 0.50312 0.01858	w=0.02108 2.64403 493.41327	
	model7	-454.782689	40	p =0.00751 q =0.00500		
	model8	-445.561588	42	p0 =0.98142 p =0.01163 q =0.00500	(p1 =0.01858) w =281.67180	54V0.998**
rps14	model0	-897.217654	39	omega (dN/dS) =0.35311		
	model1	-875.875652	40	p=0.68765 0.31235	w=0.00000 1.00000	
	model2	-872.884188	42	p=0.70412 0.23447 0.06141	w=0.01419 1.00000 3.72094	
	model3	-872.853674	43	p=0.66611 0.26357 0.07032	w=0.00000 0.82981 3.52715	

	model7	-875.895683	40	p =0.00581 q =0.01160						
	model8	-872.876409	42	p0 =0.93534 p =0.01816 q =0.05421	(p1 =0.06466) w =3.64375					
rps15	model0	-869.017168	39	omega (dN/dS) =0.31116						
	model1	-859.163668	40	p=0.74780 0.25220	w=0.12598 1.00000					
	model2	-859.163668	42	p=0.74780 0.15890 0.09330	w=0.12598 1.00000 1.00000					
	model3	-858.215082	43	p=0.35381 0.54815 0.09804	w=0.00000 0.35783 1.36915					
	model7	-858.758957	40	p =0.34292 q =0.74231						
	model8	-858.459564	42	p0 =0.92823 p =0.52609 q =1.57151	(p1 =0.07177) w =1.40061					
rps16	model0	-756.697987	39	omega (dN/dS) =1.26927						
	model1	-754.608854	40	p=0.32091 0.67909	w=0.25680 1.00000					
	model2	-744.781967	42	p=0.04677 0.74726 0.20597	w=0.00000 1.00000 7.05645	58L1.000**				
	model3	-744.856298	43	p=0.20754 0.55345 0.23901	w=0.74063 0.74064 6.11365					
	model7	-755.018833	40	p =0.45870 q =0.09475						
	model8	-744.857625	42	p0 =0.76137 p =99.00000 q =34.42408	(p1 =0.23863) w =6.11914	27E0.991**	58L1.000**			
rps18	model0	-792.007535	39	omega (dN/dS) =0.34767						
	model1	-784.899427	40	p=0.66645 0.33355	w=0.08772 1.00000					
	model2	-784.660678	42	p=0.68622 0.29270 0.02108	w=0.10334 1.00000 3.63942					
	model3	-784.682872	43	p=0.25292 0.49822 0.24886	w=0.12763 0.12763 1.35788					
	model7	-785.196811	40	p =0.16840 q =0.25837						
	model8	-784.684887	42	p0 =0.75396 p =14.83931 q =99.00000	(p1 =0.24604) w =1.36456					
rps19	model0	-478.844297	39	omega (dN/dS) =0.20515						
	model1	-477.344334	40	p=0.81619 0.18381	w=0.07325 1.00000					
	model2	-477.344278	42	p=0.81619 0.14458 0.03923	w=0.07325 1.00000 1.00000					
	model3	-477.033176	43	p=0.60431 0.24168 0.15401	w=0.00000 0.55227 0.55227					
	model7	-477.153456	40	p =0.20669 q =0.71159						
	model8	-477.153458	42	p0 =0.99999 p =0.20669 q =0.71162	(p1 =0.00001) w =1.00000					
ycf1	model0	-7063.952184	39	omega (dN/dS) =1.16459						
	model1	-6988.744955	40	p=0.40985 0.59015	w=0.16065 1.00000					
	model2	-6905.729221	42	p=0.25918 0.57477 0.16605	w=0.15673 1.00000 4.48763	44S0.998**	96Q0.999**	124R1.000**	131P1.000**	134P0.997**
						135L0.992**	55K0.994**	192P1.000**	204N1.000**	217Q1.000**
						320A0.999**	327Y0.996**	382D0.999**		
	model3	-6902.641718	43	p=0.49278 0.42248 0.08473	w=0.36036 1.78275 6.42106					
	model7	-6998.621574	40	p =0.31875 q =0.14978						

	model8	-6906.68735	42	p0 =0.82510 p =0.34857 q =0.12876	(p1 =0.17490) w =4.42845	44S0.999**	96Q0.999**	124R1.000**	131P1.000**	134P0.998**
						135L0.995**	155K0.996**	192P1.000**	204N1.000**	217Q1.000**
						313R0.992**	314P0.990**	320A1.000**	327Y0.998**	382D0.999**
ycf2	model0	-8911.80907	39	omega (dN/dS) =0.88577						
	model1	-8882.989788	40	p=0.35811 0.64189	w=0.00000 1.00000					
	model2	-8858.616609	42	p=0.31905 0.60694 0.07401	w=0.00000 1.00000 5.25880	159R0.995**	278I0.998**			
	model3	-8857.038358	43	p=0.51029 0.47309 0.01662	w=0.10036 1.71918 10.42651					
	model7	-8883.686541	40	p =0.00763 q =0.00500						
	model8	-8858.827955	42	p0 =0.91518 p =0.03033 q =0.01692	(p1 =0.08482) w =4.92112	159R0.997**	277K0.993**	278I0.999**		
ycf3	model0	-1223.692567	39	omega (dN/dS) =0.12214						
	model1	-1223.692634	40	p=0.99999 0.00001	w=0.12214 1.00000					
	model2	-1224.135529	42	p=1.00000 0.00000 0.00000	w=0.12487 1.00000 1.00000					
	model3	-1223.692444	43	p=0.22666 0.26656 0.50678	w=0.12214 0.12214 0.12214					
	model7	-1223.716232	40	p =13.87727 q =99.00000						
	model8	-1223.716437	42	p0 =0.99999 p =13.87682 q =99.00000	(p1 =0.00001) w =1.00000					
ycf4	model0	-898.191403	39	omega (dN/dS) =0.93575						
	model1	-889.319486	40	p=0.40657 0.59343	w=0.00000 1.00000					
	model2	-884.496114	42	p=0.67597 0.00000 0.32403	w=0.23210 1.00000 2.71664					
	model3	-884.496114	43	p=0.16191 0.51405 0.32403	w=0.23210 0.23210 2.71664					
	model7	-889.322752	40	p =0.00848 q =0.00561						
	model8	-884.499812	42	p0 =0.67665 p =30.21125 q =99.00000	(p1 =0.32335) w =2.71983	30R0.992**				

Table S7. Likelihood ratio test (LRT) of the variable ω ratio under different models.

gene	comparisons	2Δl		df	p
accD	M0 vs M3	237.966698	118.983349	4	8.81E-25
	M1 vs M2	85.152726	42.576363	2	5.68E-10
	M7 vs M8	91.184942	45.592471	2	1.26E-10
ccsA	M0 vs M3	43.479176	21.739588	4	2.26E-04
	M1 vs M2	18.233124	9.116562	2	1.05E-02
	M7 vs M8	20.41827	10.209135	2	6.07E-03
petD	M0 vs M3	91.556788	45.778394	4	2.74E-09
	M1 vs M2	3.768938	1.884469	2	3.90E-01
	M7 vs M8	5.182296	2.591148	2	2.74E-01
petL	M0 vs M3	2.180144	1.090072	4	8.96E-01
	M1 vs M2	1.27689	0.638445	2	7.27E-01
	M7 vs M8	2.749426	1.374713	2	5.03E-01
psbH	M0 vs M3	99.1535	49.57675	4	4.43E-10
	M1 vs M2	57.516426	28.758213	2	5.69E-07
	M7 vs M8	57.488446	28.744223	2	5.73E-07
rbcL	M0 vs M3	230.978146	115.489073	4	4.91E-24
	M1 vs M2	41.00859	20.504295	2	3.53E-05
	M7 vs M8	48.134632	24.067316	2	5.94E-06
rpl22	M0 vs M3	82.36515	41.182575	4	2.46E-08
	M1 vs M2	14.537126	7.268563	2	2.64E-02
	M7 vs M8	16.053856	8.026928	2	1.81E-02
rpl32	M0 vs M3	37.381052	18.690526	4	9.04E-04
	M1 vs M2	15.298988	7.649494	2	2.18E-02
	M7 vs M8	16.997718	8.498859	2	1.43E-02
rpoC1	M0 vs M3	191.648886	95.824443	4	7.61E-20
	M1 vs M2	29.153216	14.576608	2	6.83E-04
	M7 vs M8	39.448928	19.724464	2	5.21E-05
rpoC2	M0 vs M3	462.624582	231.312291	4	6.89E-49
	M1 vs M2	52.177988	26.088994	2	2.16E-06
	M7 vs M8	79.29341	39.646705	2	2.46E-09
rps12	M0 vs M3	31.240522	15.620261	4	3.57E-03
	M1 vs M2	18.615056	9.307528	2	9.53E-03
	M7 vs M8	18.442202	9.221101	2	9.95E-03
rps15	M0 vs M3	21.604172	10.802086	4	4.51E-03
	M1 vs M2	0	0	2	1.00E+00
	M7 vs M8	0.598786	0.299393	2	8.61E-01
rps16	M0 vs M3	23.683378	11.841689	4	1.86E-02
	M1 vs M2	19.653774	9.826887	2	7.35E-03
	M7 vs M8	20.322416	10.161208	2	6.22E-03
ycf1	M0 vs M3	322.620932	161.310466	4	7.65E-34
	M1 vs M2	166.031468	83.015734	2	9.41E-19
	M7 vs M8	183.868448	91.934224	2	1.09E-20

ycf2	M0 vs M3	109.541424	54.770712	4	3.63E-11
	M1 vs M2	48.746358	24.373179	2	5.10E-06
	M7 vs M8	49.717172	24.858586	2	4.00E-06
ycf4	M0 vs M3	27.390578	13.695289	4	8.33E-03
	M1 vs M2	9.646744	4.823372	2	8.97E-02
	M7 vs M8	9.64588	4.82294	2	8.97E-02
